# A mathematically rigorous algorithm to define, compute and assess relevance of the probable dissociation constants in characterizing a biochemical network

**DOI:** 10.1038/s41598-024-53231-9

**Published:** 2024-02-12

**Authors:** Siddhartha Kundu

**Affiliations:** Department of Biochemistry, All India Institute of Medical Sciences, Ansari Nagar, New Delhi, 110029 India

**Keywords:** Biochemistry, Computational biology and bioinformatics, Systems biology, Mathematics and computing

## Abstract

Metabolism results from enzymatic- and non-enzymatic interactions of several molecules, is easily parameterized with the dissociation constant and occurs via biochemical networks. The dissociation constant is an empirically determined parameter and cannot be used directly to investigate in silico models of biochemical networks. Here, we develop and present an algorithm to define, compute and assess the relevance of the probable dissociation constant for every reaction of a biochemical network. The reactants and reactions of this network are modelled by a stoichiometry number matrix. The algorithm computes the null space and then serially generates subspaces by combinatorially summing the spanning vectors that are non-trivial and unique. This is done until the terms of each row either monotonically diverge or form an alternating sequence whose terms can be partitioned into subsets with almost the same number of oppositely signed terms. For a selected null space-generated subspace the algorithm utilizes several statistical and mathematical descriptors to select and bin terms from each row into distinct outcome-specific subsets. The terms of each subset are summed, mapped to the real-valued open interval $\left(0,\infty \right)$ and used to populate a reaction-specific outcome vector. The p1-norm for this vector is then the probable dissociation constant for this reaction. These steps are continued until every reaction of a modelled network is unambiguously annotated. The assertions presented are complemented by computational studies of a biochemical network for aerobic glycolysis. The fundamental premise of this work is that every row of a null space-generated subspace is a valid reaction and can therefore, be modelled as a reaction-specific sequence vector with a dimension that corresponds to the cardinality of the subspace after excluding all trivial- and redundant-vectors. A major finding of this study is that the row-wise sum or the sum of the terms contained in each reaction-specific sequence vector is mapped unambiguously to a positive real number. This means that the probable dissociation constants, for all reactions, can be directly computed from the stoichiometry number matrix and are suitable indicators of outcome for every reaction of the modelled biochemical network. Additionally, we find that the unambiguous annotation for a biochemical network will require a minimum number of iterations and will determine computational complexity.

## Introduction

The analysis of genome- or large-scale biochemical networks is an important consideration of the “omics”- revolution and a critical step in bridging the genotype and phenotype divide. Despite the availability and accessibility of advanced data analytical tools, true mechanistic insights into the manner in which a biochemical network accomplishes complex function has remained elusive. This is partly due to the absence of a consensus on the choice of parameters needed to characterize a biochemical network. Biochemical networks are a complex interplay of nodes (genes, proteins, metabolites) and stimuli that occur in the cytosol and/or its compartments and are regulated by the laws of mass action, diffusion, feedback, partial reactions and thermodynamic parameters^1,2^. The numerical characterization of a biochemical network is based on simple assumptions in an effort to ensure rigor, maintain computational efficiency and minimize storage. Although informative, insightful and robust, the results that emerge tend to depart significantly from the intracellular milieu and are likely to be of limited biomedical relevance.

The algorithms currently deployed to investigate large biochemical networks include one or more optimization(s) and the enumeration of elementary modes. The former includes flux balance analysis (FBA), dynamic FBA, flux-variable analysis (FVA), regulatory on–off minimization (ROOM) and minimization of metabolic adjustment (MOMA)^1,3,4^. These algorithms maximize or minimize the biomass of a metabolite of interest and can be used to investigate the effects of deletions and other perturbations on the flux of metabolites through a large network^1–5^. The algorithms are computationally efficient and can be implemented with ease on an average desktop computer. However, deleting a node as in “gene deletion”-based analyses will alter the network irrevocably, a scenario which is unlikely to occur within the cell. Additionally, user-defined constraints for one or more parameters will introduce a bias into the computations and thence into the set of solutions further mitigating the impact of any analyses on true biochemical and physiological function. The enumeration of elementary flux modes (EFMs), elementary flux vectors (EFVs) and extreme pathway analysis (ExPas) is an alternative strategy to derive meaningful information from biochemical networks^2,6–9^. This set of algorithms subsumes that a single stoichiometry matrix will lead to one of several states for the “entire”-network of reactions. In these studies a network-state is modelled as a mode (norm, sign) or vector (sign) of “all” rather than individual reactions. Another fundamental assumption is that the solutions of the linear system of equations are strictly positive^6^. Here, too, since each reaction may have several outcomes, this constraint cannot be assumed de facto. The summations and subsequent selection are NP-hard and will result in voluminous run-time data^2^. Despite these limitations the algorithms can identify metabolic hubs and smaller subsets of cooperating reactions^1,2,6^.

An alternate class of algorithms is concerned with ascribing functionality to each node of a modelled biochemical network by assigning weights to all possible outcomes. The resulting causal networks are probabilistic in construction and the conclusions drawn are inferential^10^. The simple architecture notwithstanding, causal networks are able to explain the mechanism of a pharmacophore in the genesis of an adverse drug reaction or reciprocally, its efficacy along with insights into the traits that may be responsible for the same and are powerful aids in precision medicine and data-driven patient management regimes^11–16^. Operations research, where decisions need to made on the assignment, allocation and distribution of resources, often, in response to an event is another domain which has benefitted from causal networks and the evaluation of several “what if”-scenarios^17^. Biologically relevant data mining for lists of potential genes, proteins and small molecules is yet another clinically relevant application where causal networks have proven to be more than useful^18–21^. The most important limitation is however, presence of- and access to- previous and existing information. This will dictate the weights for all possible outcomes and determine the overall directionality of the biochemical network. The availability of genome-scale data has permitted investigators to analyse biochemical networks for several organisms, from single cells and with varying time points^22–24^.

The aforementioned studies (optimization, enumeration, causal networks) are exploratory, discovery-based and focussed on inferring parameters from the data itself, which is in most cases empirical. However, mixed-case scenarios where investigators combine modelling with laboratory data have gained considerable traction in recent years^24–26^. For example, there is an increased usage of laboratory assays that are based on computationally-derived structure-based molecular descriptors^27–29^. A generic strategy is to identify the core architecture of a modelled biochemical network and then generate several “synthetic”- or “redundant”-representatives which are progressively trimmed in an effort to reconstruct the originally identified structure^30^. The parameters that are inferred by this process are then numerically- and/or statistically-validated. Although the utility of inferring network-specific parameters (clustering coefficient, path distance) and data-driven (clinical, epidemiological, pharmacophore) modelling is not debatable, portability/translatability and widespread usage remain unresolved issues^25–29^. The inferred parameters, too, will be of limited utility in comprehending the underlying molecular biology and ascribing function to the modelled biochemical network. Selecting a parameter that is simultaneously biochemically and biophysically relevant, from raw datasets, is not trivial and remains a challenge. Certain parameters are truly empirical (reaction rate, rate constant, order of a reaction, turn over number) and are wholly dependent on the experimental design and setup. The inferences and thence the interpretation of these will be of limited clinical and biomedical relevance^22–26^. Hybrid descriptors such as structurally derived factors for small molecules will combine data points (empirical, computational) with complex theoretical frameworks^27–29^. It is also entirely possible to derive parameters such as dissociation constant, Michaelis–Menten constant and the Gibbs free energy change directly from theory^30–34^.

Here, we develop and present an algorithm to compute probable dissociation constants for every reaction of a biochemical network. The algorithm will utilize both, biochemically relevant and theoretically sound constraints to compute these from a stoichiometry number matrix of a biochemical network. Results will be presented, initially for a single reaction and then extended to the whole network. These studies will be complemented with the computation and analysis of a well characterized biochemical network of aerobic glycolysis (AG). The manuscript is organized into an introductory section where some of the principles and definitions that will be used by the algorithm will be defined and explained. A stepwise description of the algorithm along with the necessary mathematical analysis and the supporting computational studies will be presented as part of the “Results and discussion”. The manuscript concludes with a summary of the salient features, limitations and possible future studies which may utilize the probable dissociation constant to characterize a biochemical network. The proofs that underlie this mathematical formalism are described in detail and is presented as part of the Supplementary Material and includes definitions (D), lemmas (L), theorems (T) and corollaries (C) or are stated without a formal proof.

## Definitions, parameterization and biomedical relevance of a stoichiometry number matrix-based model for a biochemical network

Metabolism comprises reactions which are enzyme- and non-enzyme (association, disassociation, exchange)-mediated. Whilst enzyme-mediated reactions involve the transformation of one or more substrates into one or more chemically distinct products, non-enzymatic reactions will result in complex formation or disassembly.

### Terminology and concepts pertinent to reaction kinetics for use by a stoichiometry number-based model of a biochemical network

The formation of a complex is common to enzyme-mediated catalysis as well as non-catalytic cycles of associations and disassociations. We will utilize the term “reaction” throughout the manuscript to indicate this,

$$\begin{array}{ll}\mathrm{Molecular \, complex}:=\left\{\begin{array}{c}Transformative\equiv Enzyme \, mediated\\ Non-transformative\end{array}\right\}\equiv \,"{\text{reaction}}"&\quad\quad\quad\quad\quad {\text{Def}}.(1)\end{array}$$

We extend this schema to the encompassing pathway and utilize the phrase “biochemical network” to represent a set of reactants/products that participate in a set of reactions (Def. (2)). We utilize the stoichiometry number for each reactant/product to indicate the presence of a change or lack thereof, that will be brought about by a reaction (Def. (3)). Consider the reaction $\left(r\right)$ with $j$-indexed $J$-reactants and with rate $\left({R}_{r}\right)$. We will define the rate constant $\left({\lambda }_{r}\right)$ as,

$$\begin{array}{lll}& {\mathbbm{a}}_{1}{A}_{1}+{\mathbbm{a}}_{2}{A}_{2}\dots {\mathbbm{a}}_{M}{A}_{M}\stackrel{{\varvec{r}}}{\to }{\mathbbm{a}}_{M+1}{A}_{M+1}+{\mathbbm{a}}_{M+2}{A}_{M+2}\dots {\mathbbm{a}}_{M+N}{A}_{M+N}& \quad\quad\quad\quad\quad \mathrm{Reaction }(1)\\ & {\lambda }_{r}=\frac{{R}_{r}}{{[{A}_{1}]}^{{l}_{{A}_{1}}}.{[{A}_{2}]}^{{l}_{{A}_{2}}}\dots {[{A}_{M}]}^{{l}_{{A}_{M}}}}&\quad\quad\quad\quad\quad (1)\\ where,& & \\ & J=M+N;J,M,N\in {\mathbb{N}}& \quad\quad\quad\quad\quad (2)\\ -{\mathbbm{a}}_{j\le M}& :=\mathrm{Stoichiometry\, number\, for \, reactant} {A}_{j\le M}&\quad\quad\quad\quad\quad {\text{Def}}.(3{\text{a}})\\ {\mathbbm{a}}_{j>M}& :=\mathrm{Stoichiometry \, number\, for\, product} {A}_{j>M}&\quad\quad\quad\quad\quad {\text{Def}}.(3{\text{b}})\\ {l}_{{A}_{j\le M}}& :=\mathrm{Stoichiometry\, coefficient\, for\, a\, reactant} \left({A}_{j}\right) &\quad\quad\quad\quad\quad {\text{Def}}.(3{\text{c}})\\ & \in {\mathbb{R}}& \\ {\psi }_{r}& :=\mathrm{Order\, for\, a \, reaction}&\quad\quad\quad\quad \quad {\text{Def}}.(3{\text{d}})\\ & =\sum_{j=1}^{j=M}{l}_{{A}_{j\le M}}\in {\mathbb{R}}&\quad\quad\quad\quad\quad (3)\end{array}$$

The intracellular environment is extremely complex and will support several forms of reactions between the reactants/products whilst precluding others^28,33–35^. For example, the intracellular milieu or cytosol is regarded as non-Newtonian and imposes constraints on the type of reactions that may occur along with changes that effect reaction rate^34,35^. These alterations in local viscosity can occur in response to acute stressors such as fluctuations in temperature and hydrogen ion concentration. The modifications have a biochemical (trehalose, glycogen) or biophysical (cytoskeleton) basis and may function to modulate diffusion and diffusion-mediated chemical reactions in vivo or in silico^34–37^. The physiological manifestations of these will include reduced motion of macromolecules, increased solubility and phagocyte movement towards a chemotactic and noxious stimulus^36–38^. In order to retain this complexity we will use a reaction vector to model the contribution of each reactant/product. For the set of contributing ${\text{j}}$-indexed ${\text{J}}$-reactants or products,

$$\begin{array}{lll}{\text{r}}& \sim \mathbf{r}&\quad\quad\quad\quad\quad (4)\\ & \stackrel{\scriptscriptstyle{\text{def}}}{=}\left[\begin{array}{c}{\mathbbm{a}}_{1}\\ {\mathbbm{a}}_{2}\\ \vdots \\ {\mathbbm{a}}_{{\text{J}}}\end{array}\right]\subset {\mathbb{Z}}^{{\text{J}}}&\quad\quad\quad\quad\quad {\text{Def}}.(4)\\ & & \\ & \mathrm{where\, \, \, j}=\mathrm{1,2}\dots {\text{J}}={\text{M}}+{\text{N}}& \end{array}$$

#### Definition 5 (D5)

The rate constant for a reaction $\left({\uplambda }_{{\text{r}}}\right)$ is a strictly positive real scalar,

$$\begin{array}{c}\begin{array}{c}{\lambda }_{r}\in {\mathbb{R}}\cap \left(0,\infty \right)\end{array}\end{array}$$

Although a reaction may have several outcomes, we will only consider the forward-, reverse- and equivalent-outcomes in this study. Here, we define reaction “equivalence” as the uncertainty with regards to the preferred outcome for a reaction (Def. (6)). This may be due to the paucity of data on the reactants/products and/or the prevailing biophysical state of the intracellular environment where the reaction is expected to occur. In contrast, the reversibility of a reaction (forward, reverse) is a well characterized outcome.

### The dissociation constant as the parameter of choice to investigate a biochemical network

Parameterizing a chemical reaction is non-trivial and is done empirically, derived theoretically or inferred from data^23,29,33,39^. For a non-enzymatic reaction the dissociation $\left({\text{Kd}}\right)$- and association $\left({\text{Ka}}\right)$-constants are used to describe reversibility (forward, reverse) at or near equilibrium^4,5,33,40^,

$$\begin{array}{lll}{\text{Kd}}& \simeq \frac{{\uplambda }_{{{\text{r}}}_{{\text{f}}}}}{{\uplambda }_{{{\text{r}}}_{{\text{b}}}}} & \quad\quad\quad\quad\quad(5)\\ {\text{where}},& & \\ & {{\text{R}}}_{{{\text{r}}}_{{\text{f}}}}={{\text{R}}}_{{{\text{r}}}_{{\text{b}}}}&\quad\quad\quad\quad\quad (6)\\ & {\uplambda }_{{{\text{r}}}_{{\text{b}}}}\ne 0&\quad\quad\quad\quad\quad (7)\end{array}$$

The dissociation constant also allows us to describe a “non-productive” reaction which could occur when two or more reactants/products associate, form a complex and remain associated (Def. (7)). Unlike non-reacting reactants/products, the reaction commences but does not terminate. This can happen when the product of an enzyme forms a covalent linkage with the active site residues and inactivates it irreversibly^41–43^. A non-productive reaction is also observed when an inhibitor binds preferentially to the bound form of an enzyme, i.e., enzyme–substrate complex, rather than the free enzyme. This unique occurrence is referred to as “uncompetitive”-inhibition and is routinely observed in the tumor protein (*TP*)53-mediated inhibition of Glucose-6-phosphate dehydrogenase (G6PD; $\mathrm{EC }1.1.1.49$) as a well-supported mechanism for tumor suppression^43^. For a non-enzymatic reaction this implies an exceptionally high binding affinity between the reactants and/or products with the consequence that these become untenable as independent reactants/products^41–43^.

The dissociation constant is a non-negative real number $\left({\text{Kd}}\in {\mathbb{R}}\cap \left(0,\infty \right)\right.)$, can be empirically determined (surface plasma resonance, fluorescence, spectroscopic methods) or theoretically derived, and is a reliable index of reaction outcome^33,44–47^,

$$\begin{array}{lll}{\text{Forward}}:& Kd>1.0& \quad\quad\quad\quad\quad \left(8\right)\\ {\text{Reverse}}:& Kd\in \left(\mathrm{0,1.0}\right)&\quad\quad\quad\quad\quad (9)\\ {\text{Equivalent}}:& Kd\simeq 1.0& \quad\quad\quad\quad\quad(10)\\ {\text{Non}}-{\text{productive}}:& Kd\simeq 0.0&\quad\quad\quad\quad\quad (11)\end{array}$$

Since the dissociation constant is a ratio, we can use it as a functional metric to compare the rates at which each reaction of a biochemical network takes place as well as the rates of the forward- and reverse-components of a reversible reaction,

$$\begin{array}{lll}\mathrm{Consider\, the\, forward \, reactions}{:}& & \\ & {\text{if}}\left(\begin{array}{c}{{\text{Kd}}}_{{\text{i}}-1}>{{\text{Kd}}}_{{\text{i}}}>{{\text{Kd}}}_{{\text{i}}+1}\\ \mathrm{where\,} {{\text{Kd}}}_{{\text{i}}-1},{{\text{Kd}}}_{{\text{i}}},{{\text{Kd}}}_{{\text{i}}+1}\in \left(1,\infty \right)\end{array}\right)& \\ & {\text{then}}, {{\text{R}}}_{{\text{i}}-1}>{{\text{R}}}_{{\text{i}}}>{{\text{R}}}_{{\text{i}}+1}&\quad\quad\quad\quad\quad (12)\\ & & \\ \mathrm{Consider\, the\, reverse \, reactions}{:}& & \\ & {\text{if}}\left(\begin{array}{c}{{\text{Kd}}}_{{\text{i}}-1}<{{\text{Kd}}}_{{\text{i}}}<{{\text{Kd}}}_{{\text{i}}+1}\\ \mathrm{where\,} {{\text{Kd}}}_{{\text{i}}-1},{{\text{Kd}}}_{{\text{i}}},{{\text{Kd}}}_{{\text{i}}+1}\in \left(\mathrm{0,1}\right)\end{array}\right)& \\ & {\text{then}}, {{\text{R}}}_{{\text{i}}-1}>{{\text{R}}}_{{\text{i}}}>{{\text{R}}}_{{\text{i}}+1}& \quad\quad\quad\quad\quad(13)\\ & & \\ \mathrm{Consider\, the \, equivalent \, reactions}{:}& & \\ & {\text{if}}\left({{\text{Kd}}}_{{\text{i}}-1}\simeq {{\text{Kd}}}_{{\text{i}}}\simeq {{\text{Kd}}}_{{\text{i}}+1}\simeq 1.0\right)& \\ & {\text{then}},{{\text{R}}}_{{\text{i}}-1}\simeq {{\text{R}}}_{{\text{i}}}\simeq {{\text{R}}}_{{\text{i}}+1}& \quad\quad\quad\quad\quad(14)\end{array}$$

The dissociation constant is versatile, informative and biochemically relevant and is therefore, the parameter of choice to investigate a biochemical network in our study. Here, we will derive a numerical measure directly from the stoichiometry number matrix of a biochemical network which we define as the “probable dissociation constant” (Def. (8)).

### Stoichiometry number-based model of a biochemical network

We now model a biochemical network $\left(\mathcalligra{p}\right)$ as the sparse stoichiometry number matrix $\left({\mathcal{S}}_{\mathcalligra{p}}\right)$ of ${\text{J}}\times \overline{\overline{{\text{I}}}}$ dimensions $\left({\mathcal{S}}_{\mathcalligra{p}}\subset {\mathbb{Z}}^{{\text{J}}\times \overline{\overline{{\text{I}}}}}\right)$. Briefly, each column of this matrix is an ${\text{i}}$-indexed $\left({\text{i}}=\mathrm{1,2}\dots \overline{\overline{{\text{I}}}}\right)$ collection of non-trivial reaction vectors $\left({\mathbf{r}}_{1}{\mathbf{r}}_{2}\dots {\mathbf{r}}_{\overline{\overline{{\text{I}}}}}\subset {\mathbb{Z}}^{{\text{J}}}\right)$. Each row of this matrix is contributed to by ${\text{j}}$-indexed $\left({\text{j}}=\mathrm{1,2}\dots {\text{J}}\right)\mathrm{ m}$-stoichiometry numbers $\left({{\text{m}}}_{{\text{ji}}}\in {\mathbb{Z}}\right)$ of ${\text{J}}$-reactants/products across all $\overline{\overline{{\text{I}}}}$,

$$\begin{array}{lll}\mathcalligra{p}& \sim {\mathbf{r}}_{1},{\mathbf{r}}_{2}\dots {\mathbf{r}}_{{\text{i}}=\overline{\overline{{\text{I}}}}}&\quad\quad\quad\quad\quad {\text{Def}}.(9)\\ & \sim \left[\begin{array}{lll}{{\text{m}}}_{11}& \cdots & {{\text{m}}}_{1\overline{\overline{{\text{I}}}}}\\ \vdots & \ddots & \vdots \\ {{\text{m}}}_{{\text{J}}1}& \cdots & {{\text{m}}}_{{\text{J}}\overline{\overline{{\text{I}}}}}\end{array}\right]&\quad\quad\quad\quad\quad {\text{Def}}.(9{\text{a}})\\ & \sim \mathcal{S}\left(\mathcalligra{p}\right)\subset {\mathbb{Z}}^{{\text{J}}\times \overline{\overline{{\text{I}}}}}&\quad\quad\quad\quad\quad {\text{Def}}.(9{\text{b}})\\ & & \\ & {\text{where}},& \\ & {\mathbf{r}}_{{\text{i}}}\in \mathcal{R}\subset {\mathbb{Z}}^{{\text{J}}}&\quad\quad\quad\quad\quad (15)\\ & {\mathbf{r}}_{{\text{i}}}\sim \left[\begin{array}{c}{{\text{m}}}_{1}\\ {{\text{m}}}_{2}\\ \vdots \\ {{\text{m}}}_{{\text{J}}}\end{array}\right]|{{\text{m}}}_{{\text{j}}}\in {\mathbb{Z}}&\quad\quad\quad\quad\quad (16)\\ & {\mathbf{r}}_{{\text{i}}}\ne \overrightarrow{0}& \quad\quad\quad\quad\quad(17)\\ & \mathcal{R}:=\mathrm{Collection \,of \,subsets \,with\, non-trivial\, reaction \, vectors}& \quad\quad\quad\quad\quad{\text{Def}}.(9{\text{c}})\end{array}$$

### The null space-generated subspace can be used to compute the probable dissociation constant for a reaction

**Figure 1:**
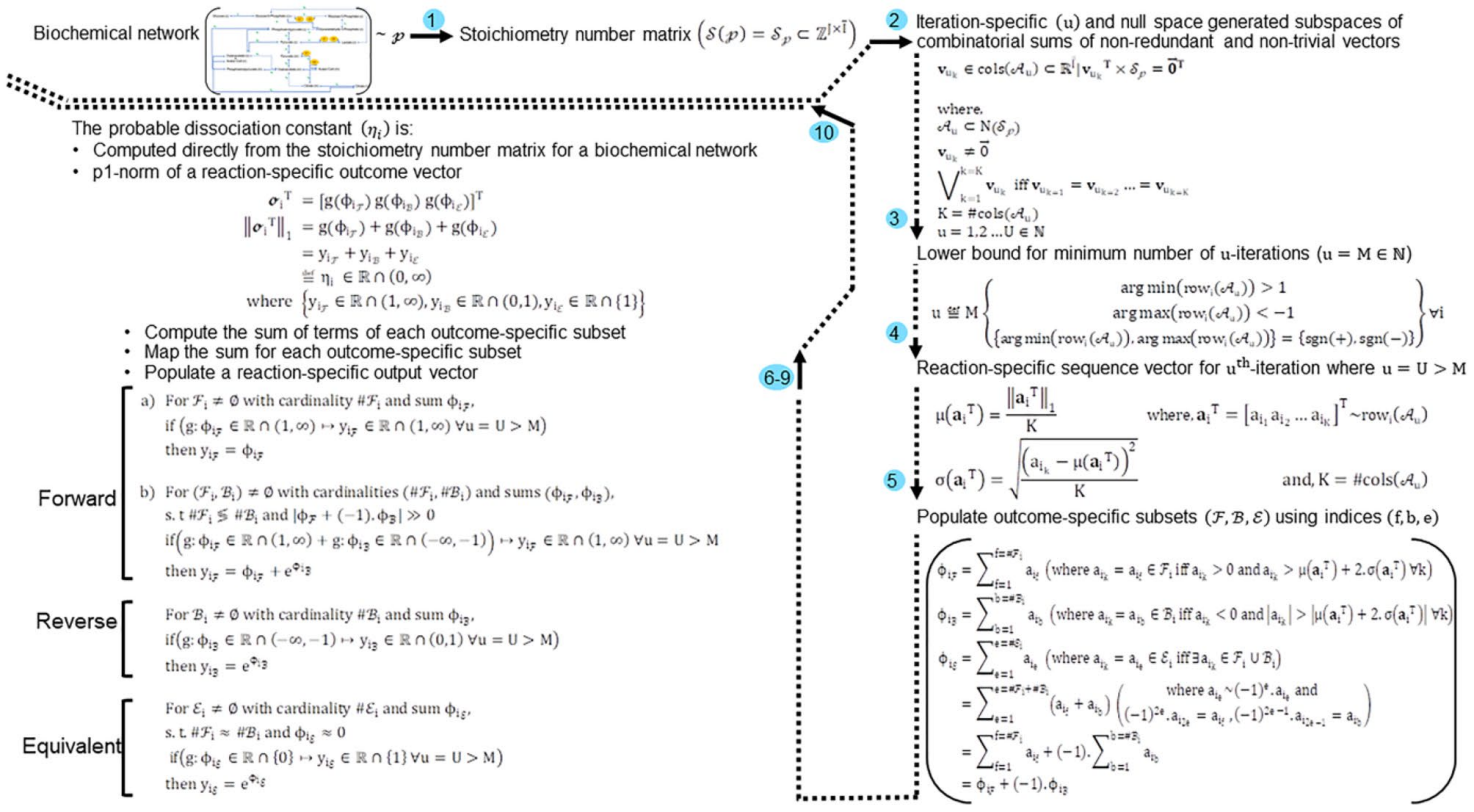
Outline of the algorithm to compute the probable dissociation constant and assign outcome to every reaction of a biochemical network. A biochemical network is modelled in terms of the stoichiometry numbers for a finite number of reactants/products and the accompanying reactions. A null space-generated subspace comprises only unique and non-trivial vectors which are iteratively, recursively and combinatorially summed. The reaction-specific sequence vector that is formed after a finite number of iterations will comprise terms that do not converge and are easily partitioned into distinct subsets. Statistical and mathematical descriptors for these terms (mean, standard deviation, greatest lower bound, least upper bound) are used to select and bin terms for all three predicted outcomes of a reaction. The formed reaction-specific outcome vector has a p1-norm which is the probable dissociation constant for a reaction. This process is repeated until every reaction of the modelled biochemical network can be unambiguously assigned. $\overline{\overline{\mathbf{I}}}$, Number of reactions for a modelled biochemical network; $\mathbf{J}$, Total number of reactants of modelled biochemical network; ${\mathcal{S}}_{\mathcalligra{p}}$, reaction-centric and user-defined stoichiometry number matrix for a biochemical network; ${\mathcal{A}}_{\mathbf{u}}$; ${\text{u}}$-iteration specific subspace of the null space of the stoichiometry number matrix for the modelled biochemical network; $\left\{{\mathcal{F}}_{{\varvec{i}}},{\mathcal{B}}_{{\varvec{i}}},{\mathcal{E}}_{{\varvec{i}}}\right\}$, Subsets to bin kth-term of an ith-reaction-specific sequence vector and ${\left({\text{u}}={\text{u}}>{\text{M}}\right)}{\text{th}}$-iteration; $\mathbf{g}(.)$; Map to assign the sum of ${\mathcal{F}}_{i}$-, ${\mathcal{B}}_{i}$- and ${\mathcal{E}}_{i}$-subsets to a strictly positive real number of an ith-reaction-specific sequence vector and ${\left({\text{u}}={\text{u}}>{\text{M}}\right)}{\text{th}}$-iteration; **T, C, D**; theorem, corollary, definition;

For a biochemical network $\left(\mathcalligra{p}\right)$ under the assumption of equilibrium, a subspace of the null space of a stoichiometry number matrix $\left(\mathcal{V}\subseteq {\text{N}}\left({\mathcal{S}}_{\mathcalligra{p}}\right)\right)$ is defined by the null space spanning vectors and those that result from their combinatorial sums (Fig. 1),

$$\begin{array}{ll}\left\{\mathbf{v}_{\text{k}}\in \mathcal{V}{\subset \mathbb{R}}^{\overline{\overline{\text{I}}}}|\begin{array}{c}\mathcal{V}\subseteq {\text{N}}\left({\mathcal{S}}_{\mathcalligra{p}}\right);{\mathbf{v}_{\text{k}}}^{\mathbf{T}}\times \mathcal{S}_{\mathcalligra{p}}={\overrightarrow{0}}^{\mathbf{T}}\end{array}\right\}&\quad\quad\quad\quad\quad {\text{Def}}.(10)\end{array}$$

$$\begin{array}{llll}{\mathcal{S}}_{\mathcalligra{p}}\subset {\mathbb{Z}}^{{\text{J}}\times \overline{\overline{{\text{I}}}}}& \stackrel{\scriptscriptstyle{\text{def}}}{=}& \mathrm{Stoichiometry \,number \,matrix\, for\, a \,biochemical\, network}& \quad\quad\quad\quad\quad(18)\\ {\text{N}}\left({\mathcal{S}}_{\mathcalligra{p}}\right)& \stackrel{\scriptscriptstyle{\text{def}}}{=}& \mathrm{Null\, space\, of }{\mathcal{S}}_{\mathcalligra{p}}& \quad\quad\quad\quad\quad(19)\\ \mathcal{V}& :=& \mathrm{Subspace\, of\, N}\left({\mathcal{S}}_{\mathcalligra{p}}\right)\mathrm{ with\, cardinality \,}\#\mathcal{V}& \quad\quad\quad\quad\quad{\text{Def}}.(11)\\ {\text{k}}& =& \mathrm{1,2}\dots \#\mathcal{V}& \quad\quad\quad\quad\quad(20)\end{array}$$

Clearly, the cardinality of a null space-generated subspace will increase with the number of cycles of combinatorial summations which we define as ${\text{u}}$-iterations (Def. (12)). The comprehensive subspace for the uth-iteration $\left({\mathcal{V}}_{{\text{u}}}\right)$ is then defined by combinatorially summing the vectors from the ${\left({\text{u}}-1\right)}$th-iteration $\left({\mathcal{V}}_{{\text{u}}-1}\right)$. Using this notation we can easily see that,

$$\begin{array}{ll}{\mathcal{V}}_{{\text{u}}=0}\sim {\text{N}}\left({\mathcal{S}}_{\mathcalligra{p}}\right)&\quad\quad\quad\quad\quad (21)\end{array}$$

#### Definition 13

A null space-generated subspace for the ${\text{uth}}$-iteration is comprehensive and defined as,

$$\begin{array}{lll}{\mathcal{V}}_{{\text{u}}}& \stackrel{\scriptscriptstyle{\text{def}}}{=}{\mathcal{V}}_{{\text{u}}=0}\cup \left({\mathcal{V}}_{{\text{u}}-1}\right)&\quad\quad\quad\quad\quad (22)\\ & ={\text{N}}\left({\mathcal{S}}_{\mathcalligra{p}}\right)\cup \left({\mathcal{V}}_{{\text{u}}-1}\right)& \quad\quad\quad\quad\quad(22.1)\\ & ={\text{N}}\left({\mathcal{S}}_{\mathcalligra{p}}\right)\cup \left({\mathcal{H}}_{{\text{u}}-1}\cup {\overline{\mathcal{H}} }_{{\text{u}}-1}\cup {\mathcal{L}}_{{\text{u}}-1}\right)& \quad\quad\quad\quad\quad(22.2)\\ {\text{where}},& & \\ {\text{N}}\left({\mathcal{S}}_{\mathcalligra{p}}\right)& =\mathrm{Null \,space \,spanning \,vectors \,for \,a \,given \,stoichiometry\, number \,matrix}& \\ {\mathcal{L}}_{{\text{u}}}& =\mathrm{ Non \,empty \,set \,of \,trivial \,vectors}& {\text{Def}}.(13{\text{a}})\\ & \stackrel{\scriptscriptstyle{\text{def}}}{=}{\mathbf{v}}_{{{\text{u}}}_{1}}={\mathbf{v}}_{{{\text{u}}}_{2}}\dots ={\mathbf{v}}_{{{\text{u}}}_{{\text{K}}}}=\overrightarrow{0}|\left\{{\mathbf{v}}_{{{\text{u}}}_{1}},{\mathbf{v}}_{{{\text{u}}}_{2}}\dots ,{\mathbf{v}}_{{{\text{u}}}_{{\text{K}}}}\right\}\in {\mathcal{L}}_{{\text{u}}}&\quad\quad\quad\quad\quad (23)\\ & \mathrm{and \,L}=\#{\mathcal{L}}_{{\text{u}}}&\quad\quad\quad\quad\quad (24)\\ {\mathcal{H}}_{{\text{u}}}& =\mathrm{Non \,empty \,set \,of \,unique\, vectors}&\quad\quad\quad\quad\quad {\text{Def}}.(13{\text{b}})\\ & \stackrel{\scriptscriptstyle{\text{def}}}{=}{\mathbf{v}}_{{{\text{u}}}_{1}}\ne {\mathbf{v}}_{{{\text{u}}}_{2}}\dots \ne {\mathbf{v}}_{{{\text{u}}}_{{\text{K}}}}|\left\{{\mathbf{v}}_{{{\text{u}}}_{1}},{\mathbf{v}}_{{{\text{u}}}_{2}}\dots ,{\mathbf{v}}_{{{\text{u}}}_{{\text{K}}}}\right\}\in {\mathcal{H}}_{{\text{u}}}&\quad\quad\quad\quad\quad (25)\\ & \mathrm{and \,H}=\#{\mathcal{H}}_{{\text{u}}}& \quad\quad\quad\quad\quad(26)\\ {\overline{\overline{\mathcal{H}}}}_{{\text{u}}}& =\mathrm{Set \,of \,identical \,vectors }&\quad\quad\quad\quad\quad {\text{Def}}.(13{\text{c}})\\ & \mathrm{where \,}\overline{\overline{{\text{H}}}}=\#{\overline{\overline{\mathcal{H}}}}_{{\text{u}}}&\quad\quad\quad\quad\quad (27)\\ & & \\ & {\text{Here}},& \\ & {\text{u}}=\mathrm{1,2}\dots .{\text{U}}\in {\mathbb{N}}& \quad\quad\quad\quad\quad(28)\end{array}$$

In order to partition ${\overline{\overline{\mathcal{H}}}}_{u}$ into subsets we will utilize the following definitions and notation,

$$\begin{array}{ll}\mathrm{Let }{{\text{A}}}_{{\text{xy}}}\subset {\overline{\overline{\mathcal{H}}}}_{{\text{u}}}&\quad\quad\quad\quad\quad {\text{Def}}.(14)\\ \mathrm{where \,x}=\#\overline{\overline{\mathcal{H}}}&\quad\quad\quad\quad\quad (29)\\ \mathrm{and \,y}=\left(\mathrm{\alpha },\upbeta ,\upgamma \right)& \quad\quad\quad\quad\quad(30)\\ & \\ \mathrm{If }\left(\mathrm{\alpha }:=\mathrm{Subset \, with \, all \, elements \, or \, with \, a \, single \, partition}\right) &\quad\quad\quad\quad\quad {\text{Def}}.(14{\text{a}})\\ \mathrm{then }\left({{\text{A}}}_{\mathrm{x\alpha }}={\overline{\overline{\mathcal{H}}}}_{{\text{u}}}\right)&\quad\quad\quad\quad\quad (30.1)\\ & \\ \mathrm{If }\left(\upbeta :=\mathrm{Subset \,with \,a \,single \,element}\right) &\quad\quad\quad\quad\quad {\text{Def}}.(14{\text{b}})\\ \mathrm{then }\left({{\text{A}}}_{\mathrm{x\beta }}\stackrel{\scriptscriptstyle{\text{def}}}{=}\left\{.\right\}\right)& \quad\quad\quad\quad\quad(30.2)\\ & \\ \mathrm{If }\left(\upgamma :=\mathrm{Subset \, with \, more \, than \, one \, element}\right) &\quad\quad\quad\quad\quad {\text{Def}}.(14{\text{c}})\\ \mathrm{then }\left({{\text{A}}}_{\mathrm{x\gamma }}\stackrel{\scriptscriptstyle{\text{def}}}{=}\left\{.,.\right\},\left\{.,.,.\right\}\dots \right)&\quad\quad\quad\quad\quad (30.3)\end{array}$$

#### Lemma 1 (L1)

*The cardinality of a subset of a finite set of identical vectors* $\left({{\text{A}}}_{{\text{xy}}}\subset \overline{\overline{\mathcal{H}}}\right)$ *is greater than unity*,

$$\begin{array}{ll}\begin{array}{c}\#{{\text{A}}}_{{\text{xy}}}>1\end{array}& \quad\quad\quad\quad\quad(31)\end{array}$$

#### Proof (L1)

By contradiction

Assume # A_xy_ = 1

$$\begin{array}{ll}& \\ \Rightarrow {{\text{A}}}_{{\text{xy}}}\subset \mathcal{H}\,({\text{unique}})&\quad\quad\quad\quad\quad (32)\\ & \end{array}$$

$$\square$$

#### Theorem 1 (T1)

*We can partition a finite set into finite non-overlapping subsets* $\left(\Theta \right)$,

$$\begin{array}{lll}\Theta & =1+{\sum }_{{\text{t}}=2}^{{\text{t}}=\#{\overline{\overline{\mathcal{H}}}}_{{\text{u}}}-2}\left(\genfrac{}{}{0pt}{}{\#{\overline{\overline{\mathcal{H}}}}_{{\text{u}}}}{{\text{t}}}\right)& \quad\quad\quad\quad\quad(33)\end{array}$$

Interestingly, we can use T1 to compute the lower bounds for a biochemical network in terms of the reactants/products $\left({\text{J}}\right)$ and reaction vectors $\left(\overline{\overline{{\text{I}}}}\right)$. Let us also assume that a single master reaction is an improbable event for a functional biochemical network given the complexity of the intracellular milieu,

$$\begin{array}{ll}{\text{if}}\left(\Theta \gg 1\right)& \\ {\text{then}}\left(\begin{array}{c}\Theta \sim {\sum }_{{\text{t}}=2}^{{\text{t}}={\text{J}}-2}\left(\genfrac{}{}{0pt}{}{{\text{J}}}{{\text{t}}}\right)\\ \stackrel{\scriptscriptstyle{\text{def}}}{=}\overline{\overline{\Theta }}\end{array}\right)&\quad\quad\quad\quad\quad (34)\end{array}$$

We see that in order to remain biochemically relevant the modelled network must have at least,

$$\begin{array}{ll}{\text{J}}\ge 4&\quad\quad\quad\quad\quad (35)\end{array}$$

The corresponding number of reaction vectors will be,

$$\begin{array}{ll}\overline{\overline{{\text{I}}}}\ge \overline{\overline{\Theta }}&\quad\quad\quad\quad\quad (36)\\ \ge 6& \quad\quad\quad\quad\quad(37)\end{array}$$

## Methods

### Computational tools to calculate the probable dissociation constant for every reaction of a biochemical network

The biochemical relevance and suitability of the probable dissociation constants for a biochemical network is illustrated by characterizing (constructed, presented, analysed) a biochemical network for AG. The R-package “ReDirection” will be used to assess the contribution of reduced- and oxidized-forms of Nicotinamide adenosine dinucleotide phosphate in regulating the activity of the Pyruvate dehydrogenase complex (PDC)^48^. “ReDirection” is freely available and can be accessed without a login id from the comprehensive R archive network (CRAN; https://cran.r-project.org/package=ReDirection)^48^.

Briefly, “ReDirection” will check and modify, if necessary, the stoichiometry number matrix for a biochemical network^48^. The matrix, along with a logical argument (TRUE, FALSE) which indicates whether the reactions are to be regarded as rows or columns, are entered as arguments for the function “*calculate_reaction_vector*”^48^. “ReDirection” will exclude all linear dependent vectors and then recheck the modified matrix for compliance with network-specific indices such as the minimum number of reactants/products, minimum number of reactions and a numerical difference of at least two in favour of the number of reactions^48^. These are necessary arguments and “ReDirection” will not process the stoichiometry number matrix if these are violated. In case there are no deficiencies, “ReDirection” will compute the null space and generate subspaces serially by combinatorially summing all non-redundant and non-trivial vectors^48^. “ReDirection” will continue these steps for a finite number of iterations until a subspace is found where the lower bounds for each sequence of row terms are unambiguous and well-defined. “ReDirection” will then check and bin every selected term to output-specific subsets (forward, reverse, equivalent), compute the linear sum for each subset, map these to strictly positive real numbers and thence populate a reaction-specific output vector^48^. The p1-norm of the latter is denoted as the probable dissociation constant for a reaction^48^. “ReDirection” will repeat this until every reaction is annotated unambiguously^48^. The output will comprise a list of reactions with their computed probable dissociation constants and the predicted outcome (forward, reverse, equivalent)^48^

### Computational studies to highlight and assess biological relevance of the probable dissociation constants

Although the probable dissociation constants that “ReDirection” computes is specific for a biochemical network, there are several generic metrics which can be derived such as the proportion and distribution of matched reactions (equivalent, non-equivalent). These can be used to indicate the flux of reactants/products in a particular direction within a network and compare the modelled biochemical network under variable intracellular conditions.

Aerobic glycolysis occurs when Glucose is converted to Lactate in the presence of non-limiting molecular dioxygen^49,50^. Although purported initially as a plausible mechanism to explain the atypical metabolism of tumorous cells, the phenomenon is routinely observed in rapidly proliferating non-tumorous (enterocytes, hematopoietic stem cells), quiescent (fibroblasts) and endothelial cells of newly forming vasculature^50^. AG offers plausible explanations for mechanisms for innate immune cell memory, angiogenesis and macrophage polarization^50–53^. For example, a common observation in pro-inflammatory polarized macrophages is a break in Kreb’s cycle at the level of citrate^54–57^. However, there is no clarity on the genesis and reversal of this outcome in the presence an intact and functioning Kreb’s cycle. Conversely, the roles of the bifunctional enzyme phosphofructokinase-2/fructose-2, 6-bisphosphatase 3 (*PFKFBP3*) in the flux of pyruvate prior to its entry into the mitochondria have been implicated in several known AG-pathways^58,59^. Central to these hypotheses is a role for the PDC and its regulation. This is a multi-enzyme ($n=3;$ Pyruvate dehydrogenase, $\mathrm{EC }1.2.4.1$; Dihydrolipoyl transacetylase, $\mathrm{EC }2.3.1.12$; Dihydrolipoyl dehydrogenase, $\mathrm{EC }1.8.1.4$) and multi-coenzyme/cofactor ($n=6$; Magnesium, Thiamine pyrophosphate, lipoic acid, coenzyme-A (CoA), ${NAD}^{+}$, flavin adenosine dinucleotide $\left({FAD}^{+}\right)$) which decarboxylates mitochondrial Pyruvate into Acetyl-CoA^60,61^. Regulation of the PDC is complex and occurs via the ratios of NAD(P)H/ NAD(P), ATP/ADP and Acetyl-CoA/CoA^60,61^.

## Results and discussion

The steady-state for a system is described as the absence of apparent change in the numbers of the reactants/products. For a biochemical network this is usually observed when the rates of formation and utilization of the reactants/products that comprise it are assumed to be equal. These conditions, when they occur within the cell are likely to be biochemical in origin and will include the effects of molecular redundancy (alternate splicing, duplicated genes, pseudogenes), feedback (positive, negative), compartmentalization and shared reactants^22,31^.

### Null space generated subspaces with reduced cardinality may improve time to complete annotation

Although the comprehensive null space-generated subspace for a modelled biochemical network is desirable, the vectors that comprise this may be trivial and redundant. This will lead to greater computational complexity and thence delay the time needed to completely annotate every reaction of a biochemical network^48^. It is therefore, imperative that these vectors are identified and excluded. The resulting subspace $\left({\mathcal{A}}_{{\text{u}}}\right)$ is of reduced cardinality and is defined as,

$$\begin{array}{ll}\left\{{\mathbf{v}}_{{{\text{u}}}_{{\text{k}}}}\in {\mathcal{A}}_{{\text{u}}}\subset {\mathcal{V}}_{{\text{u}}}{\subset {\mathbb{R}}}^{\overline{\overline{{\text{I}}}}}|{\mathcal{V}}_{{\text{u}}}\subseteq {\text{N}}\left({\mathcal{S}}_{\mathcalligra{p}}\right),\begin{array}{c}{{\mathbf{v}}_{{{\text{u}}}_{{\text{k}}}}}^{\mathbf{T}}\times {\mathcal{S}}_{\mathcalligra{p}}={\overrightarrow{0}}^{\mathbf{T}}\end{array}\right\}& \quad\quad\quad\quad\quad{\text{Def}}.(15)\end{array}$$

$$\begin{array}{lll}{\mathcal{S}}_{\mathcalligra{p}}\subset {\mathbb{Z}}^{{\text{J}}\times \overline{\overline{{\text{I}}}}}& =\mathrm{Stoichiometry\, number\, matrix\, for\, the\, biochemical \, network}& \\ {\text{N}}\left({\mathcal{S}}_{\mathcalligra{p}}\right)& =\mathrm{Null \, space \, of \, }{\mathcal{S}}_{\mathcalligra{p}}& \\ {\mathcal{V}}_{{\text{u}}}& =\mathrm{Subspace\, of\, N}\left({\mathcal{S}}_{\mathcalligra{p}}\right)\mathrm{ with \, cardinality \,}\#{\mathcal{V}}_{{\text{u}}}& \\ {\mathcal{A}}_{{\text{u}}}& =\mathrm{Null\, space\, generated \, subspace\, with\, reduced\, cardinality }& \\ {\mathbf{v}}_{{{\text{u}}}_{{\text{k}}}}\ne \overrightarrow{0}& \stackrel{\scriptscriptstyle{\text{def}}}{=}{\text{Non}}-\mathrm{trivial\, vector \, of }{\mathcal{A}}_{{\text{u}}}&\quad \quad\quad(38)\\ {\mathbf{v}}_{{{\text{u}}}_{{\text{k}}}}& \stackrel{\scriptscriptstyle{\text{def}}}{=}\mathrm{Unique \, and \, non}-\mathrm{redundant \, vector\, of }{\mathcal{A}}_{{\text{u}}}& \quad\quad\quad\quad\quad(39)\\ {\text{k}}& =\mathrm{1,2}\dots \#{\text{cols}}\left({\mathcal{A}}_{{\text{u}}}\right)&\quad\quad\quad\quad\quad (40)\\ {\text{u}}& =\mathrm{1,2}\dots {\text{U}}& \end{array}$$

The following corollaries about the cardinality of ${\mathcal{A}}_{u}$ hold and are easily established.

#### Corollary 1 (C1)

*The expected cardinality of null space generated subspace for the matrix of stoichiometric numbers of the reactants/products of a biochemical network is always greater than the computed cardinality*,

$$\begin{array}{ll}\begin{array}{c}\#{\mathcal{V}}_{{\text{u}}}>\#{\mathcal{A}}_{{\text{u}}}|{\text{u}}=\mathrm{1,2}\dots {\text{U}}\end{array}&\quad\quad\quad\quad\quad (41)\end{array}$$

#### Corollary 2 (C2; without proof)

*The cardinality of an arbitrary and recursively generated subspace of the null space of the matrix of stoichiometry numbers of the reactants/products for a biochemical network is always greater than the nullity of the null space*,

$$\begin{array}{lll}\#{\mathcal{A}}_{{\text{u}}}& =\#{\text{N}}\left({\mathcal{S}}_{\mathcalligra{p}}\right)+\left(\#{\mathcal{H}}_{{\text{u}}-1}+\#{\overline{\mathcal{H}} }_{{\text{u}}-1}+\#{\mathcal{L}}_{{\text{u}}-1}\right)& \left[\mathrm{From\, T}1,{\text{C}}1\mathrm{\, and \,Def}.(13)\right] \quad\quad\quad\quad\quad(42)\\ & =\#{\text{N}}\left({\mathcal{S}}_{\mathcalligra{p}}\right)+{\sum }_{{\text{q}}=2}^{{\text{q}}=\#{\mathcal{V}}_{{\text{u}}-1}}\left(\genfrac{}{}{0pt}{}{\#{\mathcal{V}}_{{\text{u}}-1}}{{\text{q}}}\right)& \quad\quad\quad\quad\quad(42.1)\\ \Rightarrow & >\#{\text{N}}\left({\mathcal{S}}_{\mathcalligra{p}}\right)&\quad\quad\quad\quad\quad (43)\end{array}$$

#### Corollary 3 (C3; without proof)

*The cardinality of recursively generated subspaces of the null space of the matrix of stoichiometric numbers of the reactants/products of a biochemical network forms a monotonically increasing sequence*,

$$\begin{array}{c}\begin{array}{rrr}& {\left(\#{\mathcal{A}}_{{\text{u}}}\right)}_{{\text{u}}\ge 1}\left|\#{\mathcal{A}}_{1}<\#{\mathcal{A}}_{2}\dots .<\#{\mathcal{A}}_{{\text{U}}}\right|{\text{u}}=\mathrm{1,2}\dots {\text{U}}&\quad\quad\quad\quad\quad (44)\end{array}\end{array}$$

Since the time taken to annotate every reaction of a biochemical network will depend on the combinatorial sums of all non-redundant and non-trivial null space generated subspace vectors, the cardinality of this subspace, at each iteration is an important determinant of computational complexity.

#### Corollary 4 (C4)

*The computational complexity per iteration in exponential-time* $\left({{\text{T}}}_{{\text{u}}}\right)$ *is*,

$$\begin{array}{ll}\begin{array}{c}{{\text{T}}}_{{\text{u}}}\in \left.\left(0,\mathcal{O}\left({2}^{\#{\mathcal{V}}_{{\text{u}}}}.\#{\mathcal{V}}_{{\text{u}}}\right)\right.\right]|{\text{u}}=\mathrm{1,2}\dots {\text{U}}\end{array}&\quad\quad\quad\quad\quad (45)\end{array}$$

These results suggest that as the number of null space generated subspace vectors increases there will be an increase in the number of iterations needed to unambiguously annotate a reaction. This in turn will result in an increased cardinality of each null space generated subspace. A corresponding increase in run-time will then be needed to completely annotate every reaction of a biochemical network^48^.

### Rationale to utilize the row-sum of a null space-generated subspace as the domain to compute the probable dissociation constant for a reaction

The vectors that comprise each null space-generated subspace represent a biochemical network at equilibrium where each row represents an ith-reaction and each numerical value belongs to ${\mathbb{R}}\cap \left(-\infty ,\infty \right)$. However, since the dissociation constant is a non-negative real number we seek a map to the set of strictly positive real numbers such that in the absence of empirical data we can directly compute the probable dissociation constant from the null space of the stoichiometry number matrix for a biochemical network,

$$\begin{array}{ll}{\text{g}}:\left(.\right)\in {\mathbb{R}}\cap \left(-\infty ,\infty \right)\mapsto \left(.\right)\in {\mathbb{R}}\cap \left(0,\infty \right)& \quad\quad\quad\quad\quad{\text{Def}}. (16)\end{array}$$

Let us partition the real part of the numerical values of any non-trivial and non-redundant null space generated subspace vector,

$$\begin{array}{ll}{{\text{v}}}_{{\text{ik}}}\stackrel{\scriptscriptstyle{\text{def}}}{=}{{\text{v}}}_{{\text{ikf}}}\mathrm{\, iff \, v}_{{\text{ik}}}\in {\mathbb{R}}\cap \left(0,\infty \right)&\quad\quad\quad\quad\quad (46)\\ {\mathrm{ v}}_{{\text{ik}}}\stackrel{\scriptscriptstyle{\text{def}}}{=}{{\text{v}}}_{{\text{ikb}}}\mathrm{\, iff \, v}_{{\text{ik}}}\in {\mathbb{R}}\cap \left(0,-\infty \right)&\quad\quad\quad\quad\quad (47)\\ {\mathrm{ v}}_{{\text{ik}}} \stackrel{\scriptscriptstyle{\text{def}}}{=}{{\text{v}}}_{{\text{ike}}}\mathrm{\, iff\, v}_{{\text{ik}}} \approx 0& \quad\quad\quad\quad\quad(48)\\ & \\ {\text{where}},& \\ {\text{f}}:=\mathrm{Index \,for \,a \,putative \,forward \,outcome\, predicting\, term \,}\left({\text{f}}\in {\mathbb{N}}\right)& \quad\quad\quad\quad\quad{\text{Def}}.(16{\text{a}})\\ {\text{b}}:=\mathrm{Index\, for \,a \,putative \,reverse\, outcome \,predicting\, term \,}\left({\text{b}}\in {\mathbb{N}}\right)&\quad\quad\quad\quad\quad {\text{Def}}.(16{\text{b}})\\ {\text{e}}:=\mathrm{Index \,for \,a \,putative\, equivalent \,outcome \,predicting\, term \,}\left({\text{e}}\in {\mathbb{N}}\right)& \quad\quad\quad\quad\quad{\text{Def}}.(16{\text{c}})\end{array}$$

Combining these,

$$\begin{array}{lll}{{\text{v}}}_{{\text{ik}}}& \stackrel{\scriptscriptstyle{\text{def}}}{=}{{\text{v}}}_{{\text{ikf}}} \vee {{\text{v}}}_{{\text{ikb}}} \vee {{\text{v}}}_{{\text{ike}}}& (49)\\ & \in \mathcal{A}\subset {\mathbb{R}}\cap \left(-\infty ,\infty \right)&\quad\quad\quad\quad\quad {\text{Def}}. (17)\end{array}$$

This means we can only map the following values unambiguously,

$$\begin{array}{ll}{\text{g}}\left({{\text{v}}}_{{\text{ikf}}}\right)\in {\mathbb{R}}\cap \left(1,\infty \right)& (50)\\ {\text{g}}\left({{\text{v}}}_{{\text{ikb}}}\right)\in {\mathbb{R}}\cap \left(\mathrm{0,1}\right)& \quad\quad\quad\quad\quad(51)\end{array}$$

For ${{\text{v}}}_{{\text{ik}}}\approx 0$ however, this mapping schema for an “equivalent”-reaction will not suffice since,

$$\begin{array}{ll}{{\text{v}}}_{{\text{ik}}} \approx 0\stackrel{\scriptscriptstyle{\text{def}}}{=}\left\{\begin{array}{c}``Equivalent" \left({{\text{v}}}_{{\text{ike}}}\right)\\ {``}{\text{Non-productive}}{"}\end{array}\right.& \quad\quad\quad\quad\quad(52)\end{array}$$

We can resolve this by treating each row of a null space generated subspace $\left(\mathcal{A}\right)$ as a finite series,

$$\begin{array}{ll}\upphi ={\sum }_{{\text{k}}=1}^{{\text{k}}={\text{K}}}{{\text{a}}}_{{\text{k}}}& \quad\quad\quad\quad\quad(53)\\ & \\ {\text{where}},& \\ {{\text{a}}}_{{\text{k}}}\in {\text{rows}}\left(\mathcal{A}\right)\subset {\mathbb{R}}^{1\times {\text{K}}}&\quad\quad\quad\quad\quad {\text{Def}}.(18)\\ {\text{K}}=\#{\text{cols}}\left(\mathcal{A}\right)&\quad\quad\quad\quad\quad (54)\\ {\text{k}}=\mathrm{1,2}\dots {\text{K}}&\quad\quad\quad\quad\quad (55)\end{array}$$

If we do this iteratively $\left({\text{u}}\right)$, recursively and across all columns of a null space-generated subspace $\left({\text{k}}=\mathrm{1,2}\dots {\text{K}}\right)$ and for all reactions $\left(\forall {\text{i}}\right)$, we obtain the following expression,

$$\begin{array}{lll}\upphi \sim {\upphi }_{{\text{u}}}& \sim {\upphi }_{{{\text{u}}}_{{\text{i}}}}&\quad\quad\quad\quad\quad (56)\\ & ={\sum }_{{\text{k}}=1}^{{\text{k}}=\mathbf{K}}{{\text{a}}}_{{{\text{u}}}_{{{\text{i}}}_{{\text{k}}}}}&\quad\quad\quad\quad\quad (56.1)\end{array}$$

#### Theorem 2 (T2)

*The numerical values that comprise each row of a null space generated subspace* $\left({\mathcal{A}}_{{\text{u}}}\right)$ *with positive- and negative-terms can be rewritten as an alternating sequence*,

$$\begin{array}{ll}{\left({{\text{a}}}_{{{\text{u}}}_{{{\text{i}}}_{{\text{k}}}}}\right)}_{{\text{k}}=\mathrm{1,2}\dots {\text{K}}}\sim {\left({\left(-1\right)}^{{\text{k}}}.{{\text{a}}}_{{{\text{u}}}_{{{\text{i}}}_{{\text{k}}}}}\right)}_{{\text{k}}=\mathrm{1,2}\dots {\text{K}}}&\quad\quad\quad\quad\quad (57)\\ & \\ {\text{where}},& \\ {{\text{a}}}_{{{\text{u}}}_{{{\text{i}}}_{{\text{k}}}}}\in {{\text{row}}}_{i}\left({\mathcal{A}}_{\mathbf{u}}\right)\subset {\mathbb{R}}^{1\times {\text{K}}}& \quad\quad\quad\quad\quad(58)\\ {\text{K}}=\#{\text{cols}}\left({\mathcal{A}}_{{\text{u}}}\right)&\quad\quad\quad\quad\quad (59)\\ {\text{k}}=\mathrm{1,2}\dots {\text{K}}&\quad\quad\quad\quad\quad (60)\\ {\text{i}}=\mathrm{1,2}\dots \overline{\overline{{\text{I}}}}& \quad\quad\quad\quad\quad(61)\\ {\text{u}}=\mathrm{1,2}\dots {\text{U}}\in {\mathbb{N}}&\quad\quad\quad\quad\quad (62)\end{array}$$

#### Theorem 3 (T3)

*The sum for the finite series formed by each row of a null space generated subspace can be mapped from* ${\mathbb{R}}\cap \left(-\infty ,\infty \right)$ *to* ${\mathbb{R}}\cap \left(0,\infty \right)$ *for the ith-reaction of the* *uth-iteration*,

$$\begin{array}{ll}{\text{g}}:{\upphi }_{{{\text{u}}}_{{\text{i}}}}\in {\mathbb{R}}\cap \left(-\infty ,\infty \right)\mapsto {{\text{y}}}_{{{\text{u}}}_{{\text{i}}}}\in {\mathbb{R}}\cap \left(0,\infty \right)& \quad\quad\quad\quad\quad(63)\\ & \\ {\text{where}},& \\ {\upphi }_{{{\text{u}}}_{{\text{i}}}}={\sum }_{{\text{k}}=1}^{{\text{k}}={\text{K}}}{{\text{a}}}_{{{\text{u}}}_{{{\text{i}}}_{{\text{k}}}}}& \\ {{\text{a}}}_{{{\text{u}}}_{{{\text{i}}}_{{\text{k}}}}}\in {{\text{row}}}_{{\text{i}}}\left({\mathcal{A}}_{\mathbf{u}}\right)\subset {\mathbb{R}}^{1\times {\text{K}}}& \\ & \\ {\text{and}},& \\ {\text{K}}=\#{\text{cols}}\left({\mathcal{A}}_{{\text{u}}}\right)& \\ {\text{k}}=\mathrm{1,2}\dots {\text{K}}& \\ {\text{i}}=\mathrm{1,2}\dots \overline{\overline{{\text{I}}}}& \\ {\text{u}}=\mathrm{1,2}\dots {\text{U}}\in {\mathbb{N}}& \end{array}$$

We can now utilize ${\upphi }_{{{\text{u}}}_{{\text{i}}}}$ to unambiguously compute the probable dissociation constant and assign an outcome (forward, reverse, equivalent) to the ${{\text{i}}}^{{\text{th}}}$-reaction and thence to every reaction of a biochemical network.

### Algorithm to compute the probable dissociation constant for every reaction of a biochemical network

An R-package implementation of the basic ideas that underline the presented algorithm is available for a user-defined biochemical network^48^. However, the mathematical basis for these findings and/or observations have not been addressed. Here, we rigorously investigate some of the several fundamental steps in the computation, usage and utility of the probable dissociation constant for every reaction of a biochemical network.

### Schema to screen combinatorially summed vectors for a null space-generated subspace

For each ${\text{u}}$-iteration the null space-generated subspace will be screened for trivial and redundant vectors and subsequently excluded. The non-redundant and non-trivial vectors that remain will constitute the cardinality for the ${\text{u}}$-iteration specific null space-generated subspace (Step 1, Fig. 1). The following assumptions will be valid for the presented schema,

$$\begin{array}{ll}\left\{\begin{array}{l}{\text{u}}=\mathrm{1,2}\dots {\text{U}}\\ {\text{i}}=\mathrm{1,2}\dots \overline{\overline{{\text{I}}}}\\\Theta \in {\mathbb{N}}\\ \#\overline{\overline{\mathcal{H}}}={\mathbb{K}}\end{array}\right\}& \quad\quad\quad\quad\quad(64)\end{array}$$

Rewriting the finite set of identical null space-generated subspace vectors for the ${{\text{u}}}^{{\text{th}}}$-iteration $\left({\overline{\overline{\mathcal{H}}}}_{u}\right)$ in terms of a finite collection of non-overlapping subsets $\left(\Theta \right)$,

$$\begin{array}{lll}{\overline{\overline{\mathcal{H}}}}_{{\text{u}}}& =\left\{\left({\mathbf{v}}_{{{\text{u}}}_{1}}={\mathbf{v}}_{{{\text{u}}}_{2}}\dots ={\mathbf{v}}_{{{\text{u}}}_{{\text{A}}}}\right), \left({\mathbf{v}}_{{{\text{u}}}_{{\text{A}}+1}}={\mathbf{v}}_{{{\text{u}}}_{{\text{A}}+2}}\dots ={\mathbf{v}}_{{{\text{u}}}_{{\text{B}}}}\right)\dots \left({\mathbf{v}}_{{{\text{u}}}_{{\mathbb{K}}-\left({\mathbb{K}}-1\right)}},\dots {\mathbf{v}}_{{{\text{u}}}_{{\mathbb{K}}-1}},{\mathbf{v}}_{{{\text{u}}}_{\mathbb{K}}}\right)\right\}& \quad\quad\quad\quad\quad(65)\\ & \mathrm{where }\left\{{\mathbf{v}}_{{{\text{u}}}_{1}},{\mathbf{v}}_{{{\text{u}}}_{2}}\dots ,{\mathbf{v}}_{{{\text{u}}}_{\mathbb{K}}}\right\}\in {\overline{\overline{\mathcal{H}}}}_{{\text{u}}}& \\ & & \\ & \mathrm{A \,unique\, representative\, from \,each \,subset\, is},& \\ & \left({\bigvee }_{{\text{k}}=1}^{{\text{k}}={\text{A}}}{\mathbf{v}}_{{{\text{u}}}_{{\text{k}}}},{\bigvee }_{{\text{k}}={\text{A}}+1}^{{\text{k}}={\text{B}}}{\mathbf{v}}_{{{\text{u}}}_{{\text{k}}}}\dots {\bigvee }_{{\text{k}}={\mathbb{K}}-\left({\mathbb{K}}-1\right)}^{{\text{k}}={\mathbb{K}}}{\mathbf{v}}_{{{\text{u}}}_{{\text{k}}}}\right)& \quad\quad\quad\quad\quad(66)\\ & & \\ & \mathrm{We\, will\, now \,reassign\, these \,vectors \,to \,}{\mathcal{H}}_{{\text{u}}},& \\ & \left({\bigvee }_{{\text{k}}=1}^{{\text{k}}={\text{A}}}{\mathbf{v}}_{{{\text{u}}}_{{\text{k}}}},{\bigvee }_{{\text{k}}={\text{A}}+1}^{{\text{k}}={\text{B}}}{\mathbf{v}}_{{{\text{u}}}_{{\text{k}}}}\dots {\bigvee }_{{\text{k}}={\mathbb{K}}-\left({\mathbb{K}}-1\right)}^{{\text{k}}={\mathbb{K}}}{\mathbf{v}}_{{{\text{u}}}_{{\text{k}}}}\right)\in {\mathcal{H}}_{{\text{u}}}& \quad\quad\quad\quad\quad(67)\\ & =\left({\bigvee }_{{\text{k}}=1}^{{\text{k}}={\text{A}}}{\mathbf{v}}_{{{\text{u}}}_{{\text{k}}}}\in {\mathcal{H}}_{{\text{u}}},{\bigvee }_{{\text{k}}={\text{A}}+1}^{{\text{k}}={\text{B}}}{\mathbf{v}}_{{{\text{u}}}_{{\text{k}}}}\in {\mathcal{H}}_{{\text{u}}}\dots {\bigvee }_{{\text{k}}={\mathbb{K}}-\left({\mathbb{K}}-1\right)}^{{\text{k}}={\mathbb{K}}}{\mathbf{v}}_{{{\text{u}}}_{{\text{k}}}}\in {\mathcal{H}}_{{\text{u}}}\right)&\quad\quad\quad\quad\quad (67.1)\\ & & \\ & \mathrm{and \,exclude \,these \,from \,}{\overline{\overline{\mathcal{H}}}}_{{\text{u}}}& \\ & \left({\bigvee }_{{\text{k}}=1}^{{\text{k}}={\text{A}}}{\mathbf{v}}_{{{\text{u}}}_{{\text{k}}}}\notin {\overline{\overline{\mathcal{H}}}}_{{\text{u}}},{\bigvee }_{{\text{k}}={\text{A}}+1}^{{\text{k}}={\text{B}}}{\mathbf{v}}_{{{\text{u}}}_{{\text{k}}}}\notin {\overline{\overline{\mathcal{H}}}}_{{\text{u}}}\dots {\bigvee }_{{\text{k}}={\mathbb{K}}-\left({\mathbb{K}}-1\right)}^{{\text{k}}={\mathbb{K}}}{\mathbf{v}}_{{{\text{u}}}_{{\text{k}}}}\notin {\overline{\overline{\mathcal{H}}}}_{{\text{u}}}\right)& \quad\quad\quad\quad\quad(68)\\ & =\left({\bigvee }_{{\text{k}}=1}^{{\text{k}}={\text{A}}}{\mathbf{v}}_{{{\text{u}}}_{{\text{k}}}},{\bigvee }_{{\text{k}}={\text{A}}+1}^{{\text{k}}={\text{B}}}{\mathbf{v}}_{{{\text{u}}}_{{\text{k}}}}\dots {\bigvee }_{{\text{k}}={\mathbb{K}}-\left({\mathbb{K}}-1\right)}^{{\text{k}}={\mathbb{K}}}{\mathbf{v}}_{{{\text{u}}}_{{\text{k}}}}\right)\notin {\overline{\overline{\mathcal{H}}}}_{{\text{u}}}&\quad\quad\quad\quad\quad (68.1)\end{array}$$

Let us now define a representative vector from each subset as,

$$\begin{array}{lll}{\upzeta }_{{\text{a}}}& \sim {\bigvee }_{{\text{k}}=1}^{{\text{k}}={\text{A}}}{\mathbf{v}}_{{\text{uk}}}& \\ {\upzeta }_{{\text{b}}}& \sim {\bigvee }_{{\text{k}}={\text{A}}+1}^{{\text{k}}={\text{B}}}{\mathbf{v}}_{{\text{uk}}}& \\ \vdots & \vdots & \\ {\upzeta }_{\mathbb{K}}& \sim {\bigvee }_{{\text{k}}={\mathbb{K}}-\left({\mathbb{K}}-1\right)}^{{\text{k}}={\mathbb{K}}}{\mathbf{v}}_{{\text{uk}}}& \quad\quad\quad\quad\quad{\text{Def}}.(19)\end{array}$$

Since there can be one and only one such vector for each subset, we can write,

$$\begin{array}{ll}{\zeta }_{1}={\zeta }_{2}\dots ={\zeta }_{\Theta }=1& \quad\quad\quad\quad\quad(69)\end{array}$$

The finite series formed is then numerically equal to the number of subsets that ${\overline{\overline{\mathcal{H}}}}_{{\text{u}}}$ is partitioned into,

$$\begin{array}{ll}{\sum }_{{\text{a}}=1}^{{\text{a}}=\Theta }{\zeta }_{a}=\Theta &\quad\quad\quad\quad\quad (70)\end{array}$$

These vectors will now be assigned to $\mathcal{H}$ which is the subset for all unique null space-generated subspace vectors. The incremented cardinality for the ${\text{uth}}$-iteration of this subset $\left({\overline{\overline{\mathcal{H}}}}_{u}\right)$ is,

$$\begin{array}{lll}\#{\mathcal{H}}_{{\text{u}}}& =\#{\mathcal{H}}_{{\text{u}}}+{\sum }_{{\text{a}}=1}^{{\text{a}}=\Theta }{\upzeta }_{{\text{a}}}&\quad\quad\quad\quad\quad (71)\\ & =\#{\mathcal{H}}_{{\text{u}}}+\Theta &\quad\quad\quad\quad\quad (71.1)\end{array}$$

Reciprocally, ${\overline{\overline{\mathcal{H}}}}_{{\text{u}}}$ will be correspondingly deficient in these vectors and can be written as,

$$\begin{array}{lll}{\overline{\overline{\mathcal{H}}}}_{{\text{u}}}& =\left({\mathbf{v}}_{{{\text{u}}}_{1}}={\mathbf{v}}_{{{\text{u}}}_{2}}\dots ={\mathbf{v}}_{{{\text{u}}}_{{\text{A}}-1}}\right), \left({\mathbf{v}}_{{{\text{u}}}_{{\text{A}}+1}}={\mathbf{v}}_{{{\text{u}}}_{{\text{A}}+2}}\dots ={\mathbf{v}}_{{{\text{u}}}_{{\text{B}}-1}}\right)\dots \left({\mathbf{v}}_{{{\text{u}}}_{{\mathbb{K}}-\left({\mathbb{K}}-1\right)}},\dots {\mathbf{v}}_{{{\text{u}}}_{{\mathbb{K}}-1}}\right)&\quad\quad\quad\quad\quad (72)\\ & {\text{where}}, \left\{{\mathbf{v}}_{{{\text{u}}}_{1}},{\mathbf{v}}_{{{\text{u}}}_{2}}\dots ,{\mathbf{v}}_{{{\text{u}}}_{{\mathbb{K}}-\Theta }}\right\}\in {\overline{\overline{\mathcal{H}}}}_{{\text{u}}}& \end{array}$$

The reduced cardinality for the ${{\text{u}}}^{{\text{th}}}$-iteration of ${\overline{\overline{\mathcal{H}}}}_{u}$ is,

$$\begin{array}{lll}\#{\mathcal{H}}_{{\text{u}}}& ={\text{A}}-1+{\text{B}}-1\dots .{\mathbb{K}}-1&\quad\quad\quad\quad\quad (73)\\ & =\left({\text{A}}+{\text{B}}\dots +{\mathbb{K}}\right)-\left(1+1\dots +1\right)&\quad\quad\quad\quad\quad (73.1)\\ & =\#{\overline{\overline{\mathcal{H}}}}_{{\text{u}}}-{\sum }_{{\text{a}}=1}^{{\text{a}}=\Theta }{1}_{{\text{a}}}&\quad\quad\quad\quad\quad (73.2)\\ & =\#{\mathcal{H}}_{{\text{u}}}-{\sum }_{{\text{a}}=1}^{{\text{a}}=\Theta }{\upzeta }_{{\text{a}}}&\quad\quad\quad\quad\quad (73.3)\\ & =\#{\mathcal{H}}_{{\text{u}}}-\Theta & \quad\quad\quad\quad\quad(73.4)\end{array}$$

#### Characterizing reaction-specific sequence vectors from the rows of selected null space-generated subspaces

We have already seen that after a finite number of iterations of combinatorial summing and vector exclusion, null space-generated subspaces with reduced cardinalities will result $\left({\mathcal{A}}_{{\text{u}}}\right)$**.** Since the terms from the ${\text{ith}}$-row when taken together, i.e., across all columns of the comprising vectors $\left({\text{cols}}\left({\mathcal{A}}_{{\text{u}}}\right)\right)$ constitute the ${\text{ith}}$-reaction, we observe that $\#{\text{cols}}\left({\mathcal{A}}_{{\text{u}}}\right)$-terms can be partitioned into distinct subsets. We define ${\text{u}}={\text{M}}\in {\mathbb{N}}$ formally as the lower bound for these iterations as (Step 3; Fig. 1).

$$\begin{array}{ll}{\text{u}}\stackrel{\scriptscriptstyle{\text{def}}}{=}\mathrm{M \,iff}\left\{\begin{array}{l}\begin{array}{l}arg \,min\left({{\text{row}}}_{{\text{i}}}\left({\mathcal{A}}_{{\text{u}}}\right)\right)>1\\ arg\, max\left({{\text{row}}}_{{\text{i}}}\left({\mathcal{A}}_{{\text{u}}}\right)\right)<-1\end{array}\\ \left\{{\text{arg min}}\left({{\text{row}}}_{{\text{i}}}\left({\mathcal{A}}_{{\text{u}}}\right)\right),\mathrm{arg\, max}\left({{\text{row}}}_{{\text{i}}}\left({\mathcal{A}}_{{\text{u}}}\right)\right)\right\}=\left\{{\text{sgn}}\left(+\right),{\text{sgn}}\left(-\right)\right\}\end{array}\right\}\forall {\text{i}}&\quad\quad\quad\quad\quad (74)\\ & \\ {\text{where}},& \\ {{\text{row}}}_{{\text{i}}}\left({\mathcal{A}}_{{\text{u}}}\right)\subset {\mathbb{R}}^{1\times {\text{K}}}& \\ {\text{i}}={1,2}\dots \overline{\overline{{\text{I}}}}& \\ {\text{K}}=\#{\text{cols}}\left({\mathcal{A}}_{{\text{u}}}\right)& \end{array}$$

The numerical values for M are easily computed from the limit of the fractional optimization of two decoupled real-valued functions when evaluated as a single ratio,

$$\begin{array}{ll}\underset{{x}}{{\text{minimize}}}\left(\frac{{\text{A}}\left({\text{x}}\right)}{{\text{B}}\left({\text{x}}\right)}\right)\ge 0&\quad\quad\quad\quad\quad (75)\\ & \\ {\text{where}}& \\ {\text{A}}\left({\text{x}}\right):{\mathbb{R}}^{{\text{K}}}\mapsto {\mathbb{R}}_{+}\cap \left\{0\right\}&\quad\quad\quad\quad\quad (76)\\ {\text{B}}\left({\text{x}}\right):{\mathbb{R}}^{{\text{K}}}\mapsto {\mathbb{R}}_{+}\cap \left\{0\right\}&\quad\quad\quad\quad\quad (77)\\ {\text{K}}=\#{\text{cols}}\left({\mathcal{A}}_{{\text{u}}}\right)& \\ {\text{u}}={1,2} \ldots {\text{U}}& \end{array}$$

Since the algorithm is based on combinatorial summations, the constituent real-valued terms of a reaction-specific sequence vector will be monotonic and their linear sum will be non-zero. This implies that convergence will occur very quickly (forward, reverse). If the linear sum of the terms of the reaction-specific sequence vector is zero, this will indicate that the sequence has an almost equal number of oppositely signed terms and will not converge (equivalent). However, here too, the use of fractional optimization, decoupling and a single ratio to minimize will result in convergence and the occurrence of a limit,

$$\begin{array}{ll}\mathrm{At\, u}={\text{M}}:& \\ & \\ {\text{if}}\left({\upphi }_{{{\text{u}}}_{{\text{i}}}}\in \left(0,+\infty \right)\right)& \\ \mathrm{then }\left(\underset{{\text{x}}\to \left(+\right)\infty }{{\text{lim}}}\left(1+\frac{{{\text{e}}}^{\mathrm{arg min}\left({{\text{row}}}_{{\text{i}}}\left({\mathcal{A}}_{{\text{u}}}\right)\right)}}{{{\text{e}}}^{{\upphi }_{{{\text{u}}}_{{\text{i}}}}}}\right)=1\right)& \quad\quad\quad\quad\quad(78)\\ & \\ \mathbf{O}\mathbf{R}& \\ & \\ {\text{if}}\left({\upphi }_{{{\text{u}}}_{{\text{i}}}}\in \left(-\infty ,0\right)\right)& \\ \mathrm{then }\left(\underset{{\text{x}}\to \left(-\right)\infty }{{\text{lim}}}\left(1+\frac{{{\text{e}}}^{{\upphi }_{{{\text{u}}}_{{\text{i}}}}}}{{{\text{e}}}^{\mathrm{arg max}\left({{\text{row}}}_{{\text{i}}}\left({\mathcal{A}}_{{\text{u}}}\right)\right)}}\right)=1\right)&\quad\quad\quad\quad\quad (79)\\ & \\ \mathbf{O}\mathbf{R}& \\ & \\ {\text{if}}\left({\upphi }_{{{\text{u}}}_{{\text{i}}}}\approx 0\right)& \\ \mathrm{then }\left(\begin{array}{c}\left(\underset{{\text{x}}\to \left(+\right)\infty }{{\text{lim}}}\left(1+\frac{{{\text{e}}}^{\mathrm{arg min}\left({{\text{row}}}_{{\text{i}}}\left({\mathcal{A}}_{{\text{u}}}\right)\right)}}{{{\text{e}}}^{{\upphi }_{{{\text{u}}}_{{\text{i}}}}}}\right)\right)\vee \left(\underset{{\text{x}}\to \left(-\right)\infty }{{\text{lim}}}\left(1+\frac{{{\text{e}}}^{{\upphi }_{{{\text{u}}}_{{\text{i}}}}}}{{{\text{e}}}^{\mathrm{arg max}\left({{\text{row}}}_{{\text{i}}}\left({\mathcal{A}}_{{\text{u}}}\right)\right)}}\right)\right)\\ =\left(1\right)\vee \left(1\right)\\ =1\end{array}\right)& \quad\quad\quad\quad\quad(80)\\ & \\ {\text{where}}& \\ {\upphi }_{{{\text{u}}}_{{\text{i}}}}={\sum }_{{\text{k}}=1}^{{\text{k}}={\text{K}}}{{\text{a}}}_{{{\text{u}}}_{{{\text{i}}}_{{\text{k}}}}}& \\ {{\text{a}}}_{{{\text{u}}}_{{{\text{i}}}_{{\text{k}}}}}\in {{\text{row}}}_{{\text{i}}}\left({\mathcal{A}}_{\mathbf{u}}\right)\subset {\mathbb{R}}^{1\times {\text{K}}}& \\ {\text{K}}=\#{\text{cols}}\left({\mathcal{A}}_{{\text{u}}}\right)& \\ {\text{k}}=\mathrm{1,2}\dots {\text{K}}& \\ {\text{i}}=\mathrm{1,2}\dots \overline{\overline{{\text{I}}}}& \\ {\text{u}}=\mathrm{1,2}\dots {\text{U}}\in {\mathbb{N}}& \end{array}$$

Now that we have established the minimum number of iterations, we will proceed to define, populate and process the reaction-specific sequence vector for every reaction of the modelled biochemical network. We will use a simplified notation hereafter without loss of generality to indicate and annotate,

$$\begin{array}{ll}{\left(.\right)}_{{{\text{u}}}_{{\text{i}}}}\sim {\left(.\right)}_{{\text{i}}}& \quad\quad\quad\quad\quad(81)\\ & \\ {\text{where}},& \\ {\text{u}}=\mathrm{1,2}\dots {\text{U}}>{\text{M}}& \\ {\text{i}}=\mathrm{1,2}\dots \overline{\overline{{\text{I}}}}& \end{array}$$

We denote an ith-reaction-specific sequence vector sequence $\left({{\mathbf{a}}_{{\text{i}}}}^{{\text{T}}}\right)$ with real values which for the uth-iteration whose sum is ${\upphi }_{{\text{i}}}$ in accordance with T2 (Step 4; Fig. 1),

$$\begin{array}{lll}{{\text{row}}}_{{\text{i}}}\left({\mathcal{A}}_{{\text{u}}}\right)& \sim {{\mathbf{a}}_{{\text{i}}}}^{{\text{T}}}&\quad\quad\quad\quad\quad (82)\\ & ={\left[{{\text{a}}}_{{{\text{i}}}_{1}}{{\text{a}}}_{{{\text{i}}}_{2}}\dots {{\text{a}}}_{{{\text{i}}}_{{\text{K}}}}\right]}^{\mathbf{T}}& (82.1)\\ {\Vert {{\mathbf{a}}_{{\text{i}}}}^{{\text{T}}}\Vert }_{1}& ={\upphi }_{{\text{i}}}&\quad\quad\quad\quad\quad (83)\\ & & \\ {\text{where}},& & \\ {{\text{a}}}_{{{\text{i}}}_{{\text{k}}}}& \in {{\mathbf{a}}_{{\text{i}}}}^{{\text{T}}}\sim {{\text{row}}}_{{\text{i}}}\left({\mathcal{A}}_{{\text{u}}}\right)\subset {\mathbb{R}}^{1\times {\text{K}}}& \\ {\text{i}}& =\mathrm{1,2}\dots \overline{\overline{{\text{I}}}}& \\ {\text{K}}& =\#{\text{cols}}\left({\mathcal{A}}_{{\text{u}}}\right)& \\ {\text{k}}& =\mathrm{1,2}\dots {\text{K}}& \\ {\text{u}}& =\mathrm{1,2}\dots {\text{U}}>{\text{M}}& \end{array}$$

Once the ith-reaction specific sequence vector is defined we will characterize it using standard statistical descriptors (Step 5; Fig. 1),

$$\begin{array}{lll}\upmu \left({{\mathbf{a}}_{{\text{i}}}}^{{\text{T}}}\right)& :=\mathrm{Arithmetic \, mean \, for \, the \,}\#{\mathcal{A}}_{{\text{u}}}-\mathrm{terms \,of \,}{{\mathbf{a}}_{{\text{i}}}}^{{\text{T}}}&\quad\quad\quad\quad\quad {\text{Def}}.(20)\\ & =\frac{{\upphi }_{{\text{i}}}}{\#{\text{cols}}\left({\mathcal{A}}_{{\text{u}}}\right)}& \quad\quad\quad\quad\quad(84)\\ & =\frac{{\Vert {{\mathbf{a}}_{{\text{i}}}}^{{\text{T}}}\Vert }_{1}}{\#{\text{cols}}\left({\mathcal{A}}_{{\text{u}}}\right)}&\quad\quad\quad\quad\quad (84.1)\\ & & \\\upsigma \left({{\mathbf{a}}_{{\text{i}}}}^{{\text{T}}}\right)& :=\mathrm{Standard \, deviation\, for \,the \,}\#{\mathcal{A}}_{{\text{u}}}-\mathrm{terms \,of \,}{{\mathbf{a}}_{{\text{i}}}}^{{\text{T}}}& {\text{Def}}.(21)\\ & =\sqrt{\frac{{\left({{\text{a}}}_{{{\text{i}}}_{{\text{k}}}}-\upmu \left({{\mathbf{a}}_{{\text{i}}}}^{{\text{T}}}\right)\right)}^{2}}{\#{\text{cols}}\left({\mathcal{A}}_{{\text{u}}}\right)}}& \quad\quad\quad\quad\quad(85)\\ & & \\ & {\text{where}},& \\ & {{\text{a}}}_{{{\text{i}}}_{{\text{k}}}}\in {{\mathbf{a}}_{{\text{i}}}}^{{\text{T}}}\in {{\text{row}}}_{{\text{i}}}\left({\mathcal{A}}_{{\text{u}}}\right)\subset {\mathbb{R}}^{1\times {\text{K}}}& \\ & {\text{K}}=\#{\text{cols}}\left({\mathcal{A}}_{{\text{u}}}\right)& \\ & & \\ & {\text{and}},& \\ & {\text{k}}=\mathrm{1,2}\dots {\text{K}}& \\ & {\text{u}}=\mathrm{1,2}\dots {\text{U}}>{\text{M}}& \\ & {\text{i}}=\mathrm{1,2}\dots \overline{\overline{{\text{I}}}}& \end{array}$$

#### Rule-based population of the outcome-specific subsets for every reaction of a biochemical network

We will first define the criteria to populate the ${\mathcal{F}}_{i}$- and ${\mathcal{B}}_{i}$-subsets from the reaction-specific sequence vector for the ith-reaction of a ${\left({\text{u}}={\text{U}}>{\text{M}}\right)}{\text{th}}$-iteration,

$$\begin{array}{lll}& \left\{\begin{array}{c}{{\text{a}}}_{{{\text{i}}}_{{\text{k}}}}={{\text{a}}}_{{{\text{i}}}_{{\text{f}}}}\in {\mathcal{F}}_{i} \,iff\, {{\text{a}}}_{{{\text{i}}}_{{\text{k}}}}>\mu \left({{\mathbf{a}}_{{\text{i}}}}^{{\text{T}}}\right)+2.\sigma \left({{\mathbf{a}}_{{\text{i}}}}^{{\text{T}}}\right)\\ and \,{{\text{a}}}_{{{\text{i}}}_{{\text{k}}}}>0\end{array}\right\}& \quad\quad\quad\quad\quad{\text{Def}}.(22)\\ & \left\{\begin{array}{c}{{\text{a}}}_{{{\text{i}}}_{{\text{k}}}}={{\text{a}}}_{{{\text{i}}}_{{\text{b}}}}\in {\mathcal{B}}_{i} \,iff \left|{{\text{a}}}_{{{\text{i}}}_{{\text{k}}}}\right|>\left|\upmu \left({{\mathbf{a}}_{{\text{i}}}}^{{\text{T}}}\right)+2.\upsigma \left({{\mathbf{a}}_{{\text{i}}}}^{{\text{T}}}\right)\right|\\ and \,{{\text{a}}}_{{{\text{i}}}_{{\text{k}}}}<0\end{array}\right\}& \quad\quad\quad\quad\quad{\text{Def}}.(23)\\ & & \\ & {\text{where}},& \\ & {{\text{a}}}_{{{\text{i}}}_{{\text{k}}}}\in {{\mathbf{a}}_{{\text{i}}}}^{{\text{T}}}\in {{\text{row}}}_{{\text{i}}}\left({\mathcal{A}}_{{\text{u}}}\right)\subset {\mathbb{R}}^{1\times {\text{K}}}& \\ &\upmu \left({{\mathbf{a}}_{{\text{i}}}}^{{\text{T}}}\right)\stackrel{\scriptscriptstyle{\text{def}}}{=}\mathrm{Arithmetic \, mean \,for \,the \,}\#{\mathcal{A}}_{{\text{u}}}-\mathrm{terms \, of \,}{{\mathbf{a}}_{{\text{i}}}}^{{\text{T}}}& \\ &\upsigma \left({{\mathbf{a}}_{{\text{i}}}}^{{\text{T}}}\right)\stackrel{\scriptscriptstyle{\text{def}}}{=}\mathrm{Standard \,deviation \, for \,the \,}\#{\mathcal{A}}_{{\text{u}}}-\mathrm{terms \,of \,}{{\mathbf{a}}_{{\text{i}}}}^{{\text{T}}}& \\ & {\text{K}}=\#{\text{cols}}\left({\mathcal{A}}_{{\text{u}}}\right)& \\ & & \\ & {\text{and}},& \\ & {\text{k}}=\mathrm{1,2}\dots {\text{K}}& \\ & {\text{f}},{\text{g}}=\mathrm{1,2}\dots {\text{F}},{\text{G}}\in {\mathbb{N}}& (86)\\ & {\text{i}}=\mathrm{1,2}\dots \overline{\overline{{\text{I}}}}& \\ & {\text{u}}=\mathrm{1,2}\dots {\text{U}}>{\text{M}}& \end{array}$$

##### Corollary 5 (C5)

*If the cardinality of the reaction-specific* ${\mathcal{F}}_{{\text{i}}}$**-** and ${\mathcal{B}}_{{\text{i}}}$*-subsets is finite we can identify a unique subset* $\left({\mathcal{E}}_{{\text{i}}}\right)$ *with terms that form an alternating sequence after* ${\text{u}}={\text{U}}>{\text{M}}$-*iterations*,

$$\begin{array}{lll}{\mathcal{E}}_{{\text{i}}}& ={\left({{\text{a}}}_{{{\text{i}}}_{{\text{e}}}}\right)}_{{\text{e}}=\mathrm{1,2}\dots \#{\mathcal{E}}_{{\text{i}}}}& (87)\\ & ={\left({\left(-1\right)}^{{\text{e}}}.{{\text{a}}}_{{{\text{i}}}_{{\text{e}}}}\right)}_{{\text{e}}=\mathrm{1,2}\dots \#{\mathcal{E}}_{{\text{i}}}}& \quad\quad\quad\quad\quad(87.1)\\ & ={\left({\left(-1\right)}^{2{\text{e}}}.{{\text{a}}}_{{{\text{i}}}_{2{\text{e}}}}+{\left(-1\right)}^{2{\text{e}}-1}.{{\text{a}}}_{{{\text{i}}}_{2{\text{e}}-1}}\right)}_{{\text{e}}=\mathrm{1,2}\dots \#{\mathcal{F}}_{{\text{i}}}+\#{\mathcal{B}}_{{\text{i}}}}&\quad\quad\quad\quad\quad (87.2)\\ & {\text{where}},& \\ & {\left(-1\right)}^{2{\text{e}}}.{{\text{a}}}_{{{\text{i}}}_{2{\text{e}}}}={{\text{a}}}_{{{\text{i}}}_{{\text{f}}}}>0 & \quad\quad\quad\quad\quad(87.3)\\ & {\left(-1\right)}^{2{\text{e}}-1}.{{\text{a}}}_{{{\text{i}}}_{2{\text{e}}-1}}={{\text{a}}}_{{{\text{i}}}_{{\text{b}}}}<0& \quad\quad\quad\quad\quad(87.4)\\ & \left\{\#{\mathcal{F}}_{{\text{i}}},\#{\mathcal{B}}_{{\text{i}}}\right\}>0& \quad\quad\quad\quad\quad(87.5)\\ & & \\ & {\text{and}},& \\ & {\text{e}},{\text{f}},{\text{g}}=\mathrm{1,2}\dots {\text{E}},{\text{F}},{\text{G}}\in {\mathbb{N}}&\quad\quad\quad\quad\quad (87.6)\\ & {\text{i}}=\mathrm{1,2}\dots \overline{\overline{{\text{I}}}}& \\ & {\text{u}}=\mathrm{1,2}\dots {\text{U}}>{\text{M}}& \end{array}$$

##### Corollary 6 (C6, without proof)

*The sum of the terms of the subset that corresponds to the “equivalent”-outcome is numerically equal to zero after a large* ${\text{u}}={\text{U}}>{\text{M}}$,

$$\begin{array}{lll}{\upphi }_{{{\text{i}}}_{\mathcal{E}}}& ={\upphi }_{{{\text{i}}}_{\mathcal{F}}}+\left(-1\right).{\upphi }_{{{\text{i}}}_{\mathcal{B}}}& \quad\quad\quad\quad\quad(88)\\ & ={\upphi }_{{\text{e}}}+{\left(-1\right).\upphi }_{{\text{e}}} \left[\mathrm{From \,T}2,{\text{T}}3,{\text{C}}5\right]& \quad\quad\quad\quad\quad(88.1)\\ & \approx 0& \quad\quad\quad\quad\quad(88.2)\\ & & \\ & \mathrm{where \, for \, a \,large \, u}={\text{U}}>{\text{M}}& \\ & \#{\mathcal{F}}_{{\text{i}}}\approx \#{\mathcal{B}}_{{\text{i}}}&\quad\quad\quad\quad\quad (89)\\ & \#{\mathcal{E}}_{{\text{i}}}=\#{\mathcal{F}}_{{\text{i}}}+\#{\mathcal{B}}_{{\text{i}}}&\quad\quad\quad\quad\quad (90)\\ & & \\ & {\text{and}},& \\ & {\text{e}},{\text{f}},{\text{g}}=\mathrm{1,2}\dots {\text{E}},{\text{F}},{\text{G}}\in {\mathbb{N}}& \\ & {\text{i}}=\mathrm{1,2}\dots \overline{\overline{{\text{I}}}}& \end{array}$$

On the basis of these criteria we can populate ${\mathcal{E}}_{{\text{i}}}$ for the ith-reaction of the ${\left({\text{u}}={\text{U}}>{\text{M}}\right)}{\text{th}}$-iteration,

$$\begin{array}{ll}{{\text{a}}}_{{{\text{i}}}_{{\text{e}}}}\sim {\left(-1\right)}^{{\text{e}}}.{{\text{a}}}_{{{\text{i}}}_{{\text{e}}}}=\left\{\begin{array}{c}{\left(-1\right)}^{2{\text{e}}}.{{\text{a}}}_{{{\text{i}}}_{2{\text{e}}}}={{\text{a}}}_{{{\text{i}}}_{{\text{f}}}}\\ {\left(-1\right)}^{2{\text{e}}-1}.{{\text{a}}}_{{{\text{i}}}_{2{\text{e}}-1}}={{\text{a}}}_{{{\text{i}}}_{{\text{b}}}}\end{array}\right\}\in {\mathcal{E}}_{i}&\quad\quad\quad\quad\quad {\text{Def}}.(24)\\ {\text{where}},& \\ {\text{e}},{\text{f}},{\text{g}}=\mathrm{1,2}\dots {\text{E}},{\text{F}},{\text{G}}\in {\mathbb{N}}& \\ {\text{i}}=\mathrm{1,2}\dots \overline{\overline{{\text{I}}}}& \end{array}$$

#### Mapping the outcome-specific sum to the real-valued open interval ${\mathbb{R}}\cap \left(0,\infty \right)$

We have already identified a generic mapping schema where the lower- and upper-bounds of the reaction-specific sequence vector that comprise the real-valued terms of the null space generated subspace can be mapped to the open interval ${\mathbb{R}}\cap \left(0,\infty \right)$ (T2, T3; C5, C6) (Steps 6–9; Fig. 1). We will now define these maps formally for each outcome-specific subset,

$$\begin{array}{lll}& \mathbf{F}\mathbf{o}\mathbf{r}\mathbf{w}\mathbf{a}\mathbf{r}\mathbf{d}:& \\ {\text{a}})& \mathrm{For \,the \,non \,empty \,subset \,}{\mathcal{F}}_{{\text{i}}} \,\mathrm{ with \,cardinality \,}\#{\mathcal{F}}_{{\text{i}}}\mathrm{\, and \,sum \, }{\upphi }_{{{\text{i}}}_{\mathcal{F}}},& \\ & \mathrm{ g}:{\upphi }_{{{\text{i}}}_{\mathcal{F}}}\in {\mathbb{R}}\cap \left(1,\infty \right)\mapsto {{\text{y}}}_{{{\text{i}}}_{\mathcal{F}}}\in {\mathbb{R}}\cap \left(1,\infty \right) \forall {\text{u}}={\text{U}}>{\text{M}}&\quad\quad\quad\quad\quad (91)\\ & & \\ & {\text{where}},& \\ & {{\text{y}}}_{{{\text{i}}}_{\mathcal{F}}}={\upphi }_{{{\text{i}}}_{\mathcal{F}}}& \quad\quad\quad\quad\quad(92)\\ & & \\ {\text{b}})& \mathrm{For \, the \,non \,empty \, subsets } \,\left({\mathcal{F}}_{{\text{i}}}, {\mathcal{B}}_{{\text{i}}}\right)\mathrm{ with \,cardinalities \,}\left(\#{\mathcal{F}}_{{\text{i}}}, \,\#{\mathcal{B}}_{{\text{i}}}\right)\mathrm{ and \,sums \,}\left({\upphi }_{{{\text{i}}}_{\mathcal{F}}},{\upphi }_{{{\text{i}}}_{\mathcal{B}}}\right),& \\ & \left({\text{g}}:{\upphi }_{{{\text{i}}}_{\mathcal{F}}}\in {\mathbb{R}}\cap \left(1,\infty \right)+{\text{g}}:{\upphi }_{{{\text{i}}}_{\mathcal{B}}}\in {\mathbb{R}}\cap \left(-\infty ,-1\right)\right)\mapsto {{\text{y}}}_{{{\text{i}}}_{\mathcal{F}}}\in {\mathbb{R}}\cap \left(1,\infty \right) \forall {\text{u}}={\text{U}}>{\text{M}}& \quad\quad\quad\quad\quad(93)\\ & {\text{iff}}, & \\ & \#{\mathcal{F}}_{{\text{i}}}\lessgtr \#{\mathcal{B}}_{{\text{i}}}& \quad\quad\quad\quad\quad(94)\\ & \left|{\upphi }_{{{\text{i}}}_{\mathcal{F}}}+\left(-1\right).{\upphi }_{{{\text{i}}}_{\mathcal{B}}}\right|\gg 0&\quad\quad\quad\quad\quad (95)\\ & & \\ & {\text{where}},& \\ & {{\text{y}}}_{{{\text{i}}}_{\mathcal{F}}}={\upphi }_{{{\text{i}}}_{\mathcal{F}}}+{{\text{e}}}^{{\upphi }_{{{\text{i}}}_{\mathcal{B}}}}&\quad\quad\quad\quad\quad (96)\\ & & \\ & \mathbf{R}\mathbf{e}\mathbf{v}\mathbf{e}\mathbf{r}\mathbf{s}\mathbf{e}:& \\ & \mathrm{For\, the \,subset\, }\mathcal{B}\mathrm{\,with \,cardinality\, }\#{\mathcal{B}}_{{\text{i}}}\mathrm{ \,and\, sum \,}{\upphi }_{{{\text{i}}}_{\mathcal{B}}},& \\ & {\text{g}}:{\upphi }_{{{\text{i}}}_{\mathcal{B}}}\in {\mathbb{R}}\cap \left(-\infty ,-1\right)\mapsto {{\text{y}}}_{{{\text{i}}}_{\mathcal{B}}}\in {\mathbb{R}}\cap \left(\mathrm{0,1}\right) \forall {\text{u}}={\text{U}}>{\text{M}}&\quad\quad\quad\quad\quad (97)\\ & & \\ & {\text{where}},& \\ & {{\text{y}}}_{{{\text{i}}}_{\mathcal{B}}}={{\text{e}}}^{{\upphi }_{{{\text{i}}}_{\mathcal{B}}}}& \quad\quad\quad\quad\quad(98)\\ & & \\ & \mathbf{E}\mathbf{q}\mathbf{u}\mathbf{i}\mathbf{v}\mathbf{a}\mathbf{l}\mathbf{e}\mathbf{n}\mathbf{t}:& \\ & \mathrm{For \,the \,non \,empty \,subset }\,\mathcal{E}\mathrm{\,with \,cardinality\, }\#{\mathcal{E}}_{{\text{i}}}\,\mathrm{ and \,sum \,}{\upphi }_{{{\text{i}}}_{\mathcal{E}}},& \\ & \mathrm{ g}:{\upphi }_{{{\text{i}}}_{\mathcal{E}}}\in {\mathbb{R}}\cap \left\{0\right\}\mapsto {{\text{y}}}_{{{\text{i}}}_{\mathcal{E}}}\in {\mathbb{R}}\cap \left\{1\right\} \forall {\text{u}}={\text{U}}>{\text{M}}& \quad\quad\quad\quad\quad(99)\\ & {\text{iff}},& \\ & \#{\mathcal{F}}_{{\text{i}}}\approx \#{\mathcal{B}}_{{\text{i}}}&\quad\quad\quad\quad\quad (99.1)\\ & {\upphi }_{{{\text{i}}}_{\mathcal{E}}}\approx 0& \quad\quad\quad\quad\quad(99.2)\\ & & \\ & {\text{where}},& \\ & {{\text{y}}}_{{{\text{i}}}_{\mathcal{E}}}={{\text{e}}}^{{\upphi }_{{{\text{i}}}_{\mathcal{E}}}}&\quad\quad\quad\quad\quad (100)\end{array}$$

#### The p1-norm of the reaction-specific outcome-vector is the probable dissociation constant for that reaction

Since every reaction can have these possible outcomes, we can define for the ith-reaction of the uth-iteration where ${\text{u}}={\text{U}}>{\text{M}}$, a reaction-specific outcome vector,

$$\begin{array}{llll}{\text{g}}\left({\upphi }_{{\text{i}}}\right)\in {{\mathcal{o}}_{i}}^{\mathbf{T}}& =& {\left[{\text{g}}\left({\upphi }_{{{\text{i}}}_{\mathcal{F}}}\right)\mathrm{ g}\left({\upphi }_{{{\text{i}}}_{\mathcal{B}}}\right)\mathrm{ g}\left({\upphi }_{{{\text{i}}}_{\mathcal{E}}}\right)\right]}^{{\text{T}}}\subset {\mathbb{R}}_{+}^{1\times 3}& \quad\quad\quad\quad\quad(101)\end{array}$$

The p1-norm describes the final outcome of the ${\text{ith}}$-reaction after a finite number of ${\text{u}}={\text{U}}>{\text{M}}$-iterations (Step 10; Fig. 1),

$$\begin{array}{llll} {\Vert {{\mathcalligra{o}}_{{\text{i}}}}^{\mathbf{T}}\Vert }_{1}& =& {\text{g}}\left({\upphi }_{{{\text{i}}}_{\mathcal{F}}}\right)+{\text{g}}\left({\upphi }_{{{\text{i}}}_{\mathcal{B}}}\right)+{\text{g}}\left({\upphi }_{{{\text{i}}}_{\mathcal{E}}}\right)& \quad\quad\quad\quad\quad(102)\\ & =& {{\text{y}}}_{{{\text{i}}}_{\mathcal{F}}}+{{\text{y}}}_{{{\text{i}}}_{\mathcal{B}}}+{{\text{y}}}_{{{\text{i}}}_{\mathcal{E}}}& \quad\quad\quad\quad\quad(102.1)\\ & \stackrel{\scriptscriptstyle{\text{def}}}{=}& {\upeta }_{{\text{i}}}\in {\mathbb{R}}\cap \left(0,\infty \right)&\quad\quad\quad\quad\quad (103)\end{array}$$

We can now summarize these assertions and unambiguously annotate every reaction of a biochemical network as,

$$\begin{array}{lll}{\text{Forward}}& {\upeta }_{{\text{i}}}>1& (104)\\ {\text{Reverse}}& {\upeta }_{{\text{i}}}\in \left(\mathrm{0,1}\right)&\quad\quad\quad\quad\quad (105)\\ {\text{Equivalent}}& {\upeta }_{{\text{i}}}\simeq 1.0& \quad\quad\quad\quad\quad(106)\end{array}$$

We can easily see that the probable dissociation constant $\left({\upeta }_{{\text{i}}}\right)$ for the ith-reaction of the modelled biochemical network can be equated to the true dissociation constant $\left({{\text{Kd}}}_{{\text{i}}}\right)$,

$$\begin{array}{ll}{\upeta }_{{\text{i}}}\sim {{\text{Kd}}}_{{\text{i}}}&\quad\quad\quad\quad\quad (107)\end{array}$$

The algorithm is then iteratively applied to all other rows of a null space-generated subspace, i.e., reactions of the modelled biochemical network $\left({\text{i}}=\mathrm{1,2}\dots \overline{\overline{{\text{I}}}}\right)$ and the probable dissociation constants are computed for $\forall {\text{i}}$. If there is ambiguity in the annotation, the combinatorial summation and subsequent steps are recursively repeated^48^. Complete annotation for every reaction of a biochemical network will occur if and only if the annotation is unambiguous. A biochemical network is only regarded as being completely annotated if and only if every reaction that comprises the network is annotated unambiguously^48^.

### Biochemical relevance and suitability of the probable dissociation constant to characterize a biochemical network of aerobic glycolysis

**Figure 2:**
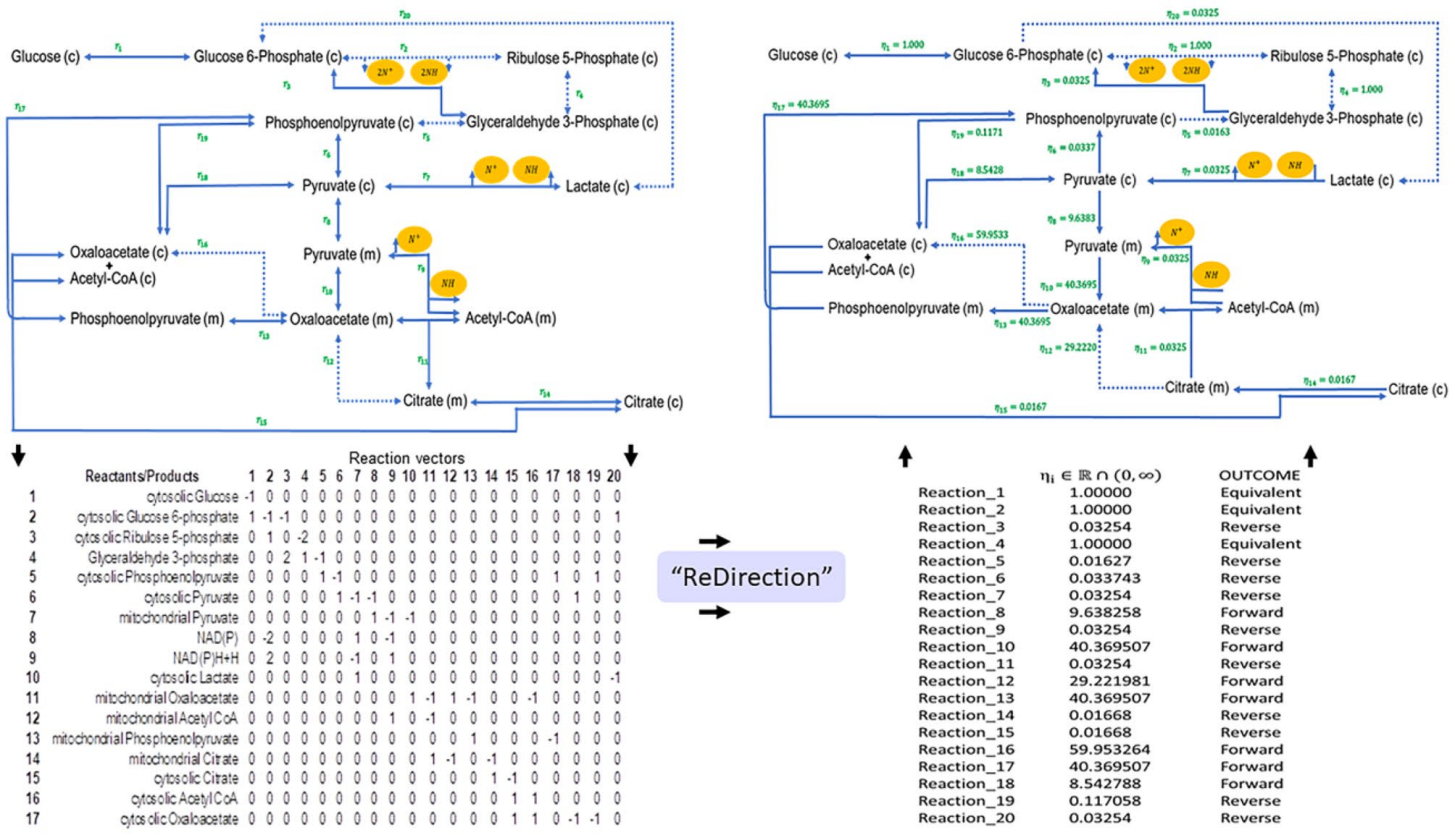
Computational studies with “ReDirection” to demonstrate suitability and biochemical relevance of the probable dissociation constant in characterizing a biochemical network for aerobic glycolysis. The dissociation constant for a reaction is an empirically derived parameter and can reveal valuable information such as the rate and rate constant for a reaction. Theoretically-derived approximations are usually based on user-defined bounds and/or pre-selecting strictly positive null space spanning vectors. Although the computations are rendered more feasible with these assumptions, the biomedical relevance of the data generated with these numeric constraints is likely to be of limited relevance. The algorithm presented computes the probable dissociation constant from first principles and with biochemically relevant constraints. A biochemical network of aerobic glycolysis is modelled and defined in terms of a sparse matrix of stoichiometric numbers of its reactants/products $\left({\text{J}}=17\right)$ and reactions $\left(\overline{\overline{{\text{I}}}}=20\right)$. “ReDirection” checks this user-defined matrix and computes the null space, utilizes mathematically sound (tests of convergence, descriptive statistics, linear maps) constraints to compute the probable dissociation constant and thereby assign a dominant direction to every reaction of the modelled biochemical network of aerobic glycolysis. $\overline{\overline{\mathbf{I}}}$, Total number of reactions of modelled biochemical network; $\mathbf{J}$, Total number of reactants of modelled biochemical network; ${\mathcal{S}}_{\mathcalligra{p}}$, reaction-centric and user-defined stoichiometry number matrix of a biochemical network for aerobic glycolysis; **m, c;** mitochondrial and cytosolic components of a reactant/product; ${{\varvec{\upeta}}}_{\mathbf{i}}$, probable dissociation constant for the ith-reaction of a biochemical network with forward $\left(\mathcal{F}\right)$, reverse $\left(\mathcal{B}\right)$, or equivalent $\left(\mathcal{E}\right)$ outcomes.

The biochemical network for AG $\left({\overline{\overline{{\text{I}}}}}_{{\text{AG}}}=20;{\upeta }_{1}-{\upeta }_{20}\right)$ comprises single (${\overline{\overline{{\text{I}}}}}_{{\text{AG}}}=14$)- and multi (${\overline{\overline{{\text{I}}}}}_{{\text{AG}}}=5$)-step reactions in the cytosolic (c) and mitochondrial (m) compartments (Fig. 2) (Supplementary Text 2). Here, the multi-step reactions include the hexose monophosphate shunt $\left({{\text{r}}}_{2},{{\text{r}}}_{4}\right)$, glycolysis $\left({{\text{r}}}_{5}\right)$, Kreb’s tricarboxylic acid (TCA; ${{\text{r}}}_{12}$), oxaloacetate-malate shuttle $\left({{\text{r}}}_{16}\right)$ and Cori’s cycle $\left({{\text{r}}}_{20}\right)$. The modelled biochemical network also includes transport reactions for Pyruvate $\left({{\text{r}}}_{8}\right)$, Citrate $\left({{\text{r}}}_{14}\right)$ and mitochondrial Phosphoenolpyruvate $\left({{\text{r}}}_{17}\right)$. The computed probable dissociation constants favour the export of oxaloacetate into the cytosol directly or through phosphophenolpyruvate $\left(\left\{{\eta }_{10},{\eta }_{13}, {\eta }_{16},{\eta }_{17}\right\}>40\right)$ thereby indicating a flux towards gluconeogenesis. The steps towards glucose 6-phosphate are supported by observing the probable dissociation constants for the following conversions: phosphoenolpyruvate $\to$ glyceraldehyde 3-phosphate $\left({\eta }_{5}\approx 0.017\right)$ and glyceraldehyde 3-phosphate $\to$ glucose 6-phosphate $\left({\eta }_{3}\approx 0.03\right)$ (Fig. 2). We can easily infer from the reverse reaction pyruvate $\to$ acetyly-CoA $\left({\eta }_{9}\approx 0.03\right)$ that the TCA-cycle is interrupted (Fig. 2). These findings suggest that contrary to available literature, the TCA-cycle is predisposed to being perturbed and its restoration or “intactness” is actively maintained for metabolic homeostasis or oxidative phosphorylation. These observations are perfectly logical given that the TCA-cycle is anaplerotic with several of its intermediates participating in collateral pathways. For AG to occur it is necessary that the TCA-cycle along with oxidative phosphorylation is interrupted in favour of an increased cell mass via the hexose monophosphate shunt pathway (DNA/RNA synthesis, NADPH) and fatty acid synthesis via acetyl-CoA (Fig. 2).

These data also offer several interesting insights into the metabolic crosstalk that may occur within cells when metabolizing Glucose to Lactate in the presence of adequate molecular dioxygen (Fig. 2). There is a significantly larger fraction $\left(85\mathrm{\%};{\overline{\overline{{\text{I}}}}}_{{\text{AG}}}=17\right)$ of non-equivalent reactions with forward $\left(35\mathrm{\%};{\overline{\overline{{\text{I}}}}}_{{\text{AG}}}=7\right)$- and reverse $\left(50\mathrm{\%};{\overline{\overline{{\text{I}}}}}_{{\text{AG}}}=10\right)$-reactions as compared to the equivalent $\left(15\mathrm{\%};{\overline{\overline{{\text{I}}}}}_{{\text{AG}}}=3\right)$ (Fig. 2). This suggests that the prevailing biochemical and physicochemical conditions where AG is expected to occur is, in fact an important determinant of whether AG occurs at all. Conversely, the paucity of equivalent reactions in the biochemical network for AG suggests lack of regulatory reactions. The ratio of ${\text{NAD}}({\text{P}}){\text{H}}$ to ${{\text{NAD}}({\text{P}})}^{+}$ along with Acetyl-CoA to CoA and ATP to ADP is an important regulator of the Pyruvate dehydrogenase complex (PDC) and is likely a major determinant for the metabolic shift observed to implement reversible AG.

### Limitations of the presented algorithm and relevance of fractional derivatives in modelling and analysing complex biochemical systems

The biomedical relevance in computing the probable dissociation constant notwithstanding, the presented algorithm is dependent on the stoichiometry or integer numbers to model change to the molecular species that comprise the modelled biochemical network. This effect is partially offset by computing the null space, although the assumption that the modelled biochemical network is at equilibrium is yet another presumption. A further limitation of the presented algorithm is that it is enumeration-based and will therefore, be intractable for large biochemical networks. Furthermore, since the computations are entirely dependent on the null space, the initial nullity is also likely to effect the real time needed to annotate every reaction of a modelled biochemical network. This will mean that for smaller networks too, the time to complete annotation may also not be possible^48^.

The probable dissociation constant is a numerical measure that is computed from the null space for a stoichiometry number matrix model of a biochemical network. However, most biochemical systems operate under non-equilibrium conditions. In fact, the true dissociation constant for a reaction is an empirical measure and is the ratio between the exponent (stoichiometry coefficients) forms for each reactant in a perfectly reversible reaction. The advent of large data warrants analytical approaches that depart significantly from previous methods in an effort to optimize computational resources whilst delivering meaningful information about the network. The use of fractional derivatives has gained traction over the last several years in an effort to impute missing data, improve convergence times and model complex systems^62–66^. For example, fractional derivatives are being used to improve convergence in the presence of multiple local optima and model and/or analyse the complex behaviour of fluids, biomolecules and biochemical systems such as anomalous diffusion in the cytoplasm and across membranes and even telomeres in the nucleoplasm^62–66^. All these studies have reported significant improvements in predicting an outcome, goodness-of-fit studies and time to convergence.

In general, a fractional derivative of $\alpha$-order can be approximated with the gamma function using the Riemann–Liouville (RL), Caputo (CAP) or the Grunwald–Letnikov (GL) methods^67–70^. Consider the Caputo fractional derivative,

$$\begin{array}{ll}{{\text{D}}}^{\mathrm{\alpha }}{{\text{x}}}^{\beta }=\left(\frac{\Gamma \left(\upbeta +1\right)}{\Gamma \left(\upbeta -\mathrm{\alpha }+1\right)}\right).{{\text{x}}}^{\upbeta -\mathrm{\alpha }}&\quad\quad\quad\quad\quad (108)\\ {\text{where}},& \\\upbeta \in {\mathbb{Z}}_{+}\cup \left\{0\right\}&\quad\quad\quad\quad\quad (109)\\ \alpha \in \left(\beta -1,\beta \right)& \quad\quad\quad\quad\quad(110)\\ \alpha \in {\mathbb{R}}_{+}& \quad\quad\quad\quad\quad(111)\end{array}$$

In the absence of empirical data investigating a biochemical network is challenging. One strategy for interrogating a biochemical network at non-equilibrium or near steady-state conditions is approximating the dissociation constant for a reaction. This equation is fundamental to biochemical analysis and involves a phenomenological approximation of the integer values for the stoichiometry numbers of the reactants with an expression that involves real number exponents for each reactant of a fully reversible reaction. In accordance with Reaction 1 this is written as,

$$\begin{array}{ll}{{\text{Kd}}}_{{\text{i}}}=\frac{{\prod }_{{\text{j}}=1}^{{\text{j}}={\text{M}}}{\left[{{\text{A}}}_{{\text{j}}}\right]}^{{\mathbbm{a}}_{{\text{j}}}}}{{\prod }_{{\text{j}}=1}^{{\text{j}}={\text{J}}-{\text{M}}}{\left[{{\text{A}}}_{{\text{j}}}\right]}^{{\mathbbm{a}}_{{\text{j}}}}}&\quad\quad\quad\quad\quad (112)\\ & \\ {\text{where}},& \\ {\mathbbm{a}}_{{\text{j}}}\in {\mathbb{R}}& \end{array}$$

We can then proceed to compute the fractional gradient descent, post hoc, to a preliminary imputation step to estimate missing or latent data points^62,63,69,70^. We can combine a small learning rate with a constant (stochastic) or mini-batch update protocol concomitantly with several novel algorithmic approaches to accomplish these steps^62,63,69,70^,

$$\begin{array}{ll}{\mathbf{r}}_{{\text{i}},{\text{x}}+1}={\mathbf{r}}_{{\text{i}},{\text{x}}}-\upgamma .{{\text{D}}}^{\mathrm{\alpha }}\left({\left({\mathbf{r}}_{{\text{i}},{\text{x}}}\right)}^{\upbeta };\left(\left({\text{x}}+1\right)\vee \left({\text{x}}+{\text{y}}\right)\right)\right)& \quad\quad\quad\quad\quad(113)\\ & \\ {\text{where}},& \\\upgamma :=\mathrm{Learning \,rate}& \\ \in {\mathbb{R}}\cap \left(\mathrm{0,1}\right)& \quad\quad\quad\quad\quad(114)\\ {\text{x}}:=\mathrm{Finite\, number\, of \,iterations}& \\ =\left(\mathrm{0,1},\dots \right)&\quad\quad\quad\quad\quad (115)\\ {\text{y}}:=\mathrm{Finite \,number\, of \,updates }& \\ \in {\mathbb{N}}& \quad\quad\quad\quad\quad(116)\end{array}$$

The imputed values computed earlier will represent an initial approximation to the original stoichiometry numbers matrix for a biochemical network and can be refined iteratively,

$$\begin{array}{ll}{{\text{D}}}^{\mathrm{\alpha }}{\left({\mathbf{r}}_{{\text{i}}}\right)}^{\upbeta }\stackrel{\scriptscriptstyle{\text{def}}}{=}{{\text{D}}}^{\mathrm{\alpha }}{\left[\begin{array}{c}{{\text{m}}}_{1}\\ {{\text{m}}}_{1}\\ \vdots \\ {{\text{m}}}_{{\text{J}}}\end{array}\right]}^{{\varvec{\upbeta}}}& \quad\quad\quad\quad\quad(117)\\ =\left[\begin{array}{c}{\frac{{\partial }^{\mathrm{\alpha }}}{\partial {{\text{m}}}_{1}}\left({{\text{m}}}_{1}\right)}^{\upbeta }\\ {\frac{{\partial }^{\mathrm{\alpha }}}{\partial {{\text{m}}}_{2}}\left({{\text{m}}}_{2}\right)}^{\upbeta }\\ \vdots \\ {\frac{{\partial }^{\mathrm{\alpha }}}{\partial {{\text{m}}}_{{\text{J}}}}\left({{\text{m}}}_{{\text{J}}}\right)}^{\upbeta }\end{array}\right]&\quad\quad\quad\quad\quad (117.1)\\ =\left[\begin{array}{c}\left(\frac{\Gamma \left(\upbeta +1\right)}{\Gamma \left(\upbeta -\mathrm{\alpha }+1\right)}\right).{\left({{\text{m}}}_{1}\right)}^{\upbeta -\mathrm{\alpha }}\\ \left(\frac{\Gamma \left(\upbeta +1\right)}{\Gamma \left(\upbeta -\mathrm{\alpha }+1\right)}\right).{\left({{\text{m}}}_{2}\right)}^{\upbeta -\mathrm{\alpha }}\\ \vdots \\ \left(\frac{\Gamma \left(\upbeta +1\right)}{\Gamma \left(\upbeta -\mathrm{\alpha }+1\right)}\right).{\left({{\text{m}}}_{{\text{J}}}\right)}^{\upbeta -\mathrm{\alpha }}\end{array}\right]& \quad\quad\quad\quad\quad(117.2)\\ \stackrel{\scriptscriptstyle{\text{def}}}{=}\left[\begin{array}{c}{\upomega }_{1}\\ {\upomega }_{2}\\ \vdots \\ {\upomega }_{{\text{J}}}\end{array}\right]&\quad\quad\quad\quad\quad (118)\\ ={\mathbf{w}}_{{\text{i}}}& (119)\\ & \\ {\text{where}},& \\ {\mathbf{r}}_{{\text{i}}}\in {\mathbb{Z}}^{{\text{J}}}& \\ {\upomega }_{{\text{j}}}\in {\mathbf{w}}_{{\text{i}}}\in {\mathbb{R}}^{{\text{J}}}&\quad\quad\quad\quad\quad (120)\\ {\text{i}}\in \left[1,\overline{\overline{{\text{I}}}}\right]& \\\upbeta \in {\mathbb{Z}}_{+}\cup \left\{0\right\}& \\ \mathrm{\alpha }\in \left(\upbeta -1,\upbeta \right)& \\ \mathrm{\alpha }\in {\mathbb{R}}_{+}& \end{array}$$

The computed numerical values can be used to assess the contribution of each molecular species to a reaction vector and are easily mapped to the exponents for each reactant. This can then be directly included in the formula to estimate the dissociation constant for a reaction at a particular step. Since this is a strictly positive real number we can use this metric to check whether each reaction vector converges to the expected outcome,

$$\begin{array}{ll}{{\text{Kd}}}_{{\text{i}}}=\frac{{\prod }_{{\text{j}}=1}^{{\text{j}}={\text{M}}}{\left[{{\text{A}}}_{{\text{j}}}\right]}^{{\upomega }_{{\text{j}}}}}{{\prod }_{{\text{j}}=1}^{{\text{j}}={\text{J}}-{\text{M}}}{\left[{{\text{A}}}_{{\text{j}}}\right]}^{{\upomega }_{{\text{j}}}}}&\quad\quad\quad\quad\quad (121)\\ ={\mathbb{R}}\cap \left(\left(\mathrm{0,1}\right)\vee \left\{1\right\}\vee \left(1,\infty \right)\right)& \quad\quad\quad\quad\quad(121.1)\end{array}$$

### Biomedical relevance of the presented algorithm to the study of biochemical networks

The dissociation constant for a reaction is a fundamental parameter in biochemical analysis and is computed predominantly from empirical data. However, this information is sparse and unavailable for several biochemical reactions of interest. For those reactions that laboratory data is available, the absence of corroborating in vivo details will render these less likely to have clinical- or biomedical-relevance. Additionally, biochemical assays comprise single or at most coupled reactions which further limits the utility of this information since complex biochemical function is brought about by biochemical networks. Although several algorithms utilize the null space, these are directed towards comprehending possible stable states for a modelled biochemical network. Parameter selection is also left to the discretion of the user further limiting the portability of these studies.

Unlike these investigations the probable dissociation constants are computed for every reaction and directly from a null space-generated subspace of the stoichiometry number matrix for a biochemical network. The algorithm concentrates on using combinatorial summations of unique and non-trivial null space spanning vectors to estimate the probable dissociation constant for every reaction along with mathematically rigorous and statistically relevant term-selection. A significant contribution of this algorithm is that it does not exclude numerical values and in doing so ensures that the computations are all encompassing. The computation of the probable dissociation constants are not only user-independent and computed from first principles, but may also be biochemically relevant. Since the mapping of the probable dissociation constants to the positive real number space is in accordance with established biochemical paradigms, the information gleaned can easily be incorporated into simulation studies for a biochemical network and used to compute the trajectories for a reactant/product^38,40^. Furthermore, the distribution of the probable dissociation constants across a biochemical network can offer plausible explanations into metabolite flux and usage. These testable hypotheses may be corroborated with detailed site-directed mutagenesis experiments and labelling studies among several others^44–47^. The probable dissociation constants can also be used to compare individual biochemical reactions of a network. These investigations may also be complemented by detailed theoretical studies such as phylogenetic analysis for enzyme-mediated reactions to ascertain the isoforms that may be deployed by a biochemical network. It may also be possible to perturb these networks and investigate the distribution for each pair of reactions of a biochemical network, i.e., before and after a stimulus.

## Conclusions

The probable disassociation constant for a biochemical reaction is a numerical measure which can be computed directly from a null space-generated subspace of the stoichiometry number matrix for a biochemical network. Here, we present a mathematically rigorous algorithm to compute the probable dissociation constant for a reaction and thence for every reaction of a biochemical network. Each step of the algorithm is outlined and supported by the necessary mathematical formalism that is needed to comprehend and utilize this framework. The theoretical assertions presented in the main text are supported by formal proofs wherever applicable and are available and accessible as Supplementary Material. The biological relevance of this work is illustrated with computational studies and analyses of the probable dissociation constants for a well characterized biochemical network. The data generated is in accordance with established kinetic paradigms and is therefore, a suitable index of biochemical function and can be used to parameterize and study a biochemical network. These data are also amenable to detailed analyses and can be used to test various hypotheses for both, baseline- and perturbed-conditions of different cell types and across taxa. Additionally, the resulting data may offer valuable insights into the physiological and thence the pathological basis of diseases such as malignancies, altered immune response, etc.

### Supplementary Information

Supplementary Information 1.

Supplementary Information 2.

## Data Availability

All data generated or analysed during this study are included in this published article [and its supplementary information files].
