# Supplementary material for: A mathematically rigorous algorithm to define, compute and assess relevance of the probable dissociation constants in characterizing a biochemical network
Source: Sci Rep. 2024 Feb 12;14:3507. doi: 10.1038/s41598-024-53231-9 (PMC10861591; doi:10.1038/s41598-024-53231-9)
Supplement: Supplementary file 1 — Supplementary Information 1. [file 41598_2024_53231_MOESM1_ESM.docx]

**Detailed proofs of definitions, theorems, lemmas and corollaries to compute the probable dissociation constant and assign outcome to every reaction of a constrained biochemical network**

The mathematical rigor of the algorithm results in a numerically robust computation of the probable dissociation constant for every reaction of a constrained biochemical network. The mathematical formalism not only adds rigor but is also necessary to parameterize and delineate the structure of the biochemical network under study.

**Proof (D5):**

Assume $\lambda_{r}$ is a vector and can be resolved into forward- $\left( \lambda_{r_{f}} \right)$ and reverse-$\left( \lambda_{r_{b}} \right)$ components,

$$\begin{matrix} \vec{\lambda}_{r} & =\left| \vec{\lambda}_{r} \right|.cos\left( \theta\right)\hat{i}+\left| \vec{\lambda}_{r} \right|.cos\left( 90-\theta\right)\hat{j} & (1) \\ & =\left| \vec{\lambda}_{r} \right|.cos\left( \theta\right)\hat{i}+\left| \vec{\lambda}_{r} \right|.sin\left( \theta\right)\hat{j} & (1.1) \\ Let, & \left| \vec{\lambda}_{r_{f}} \right|=\left| \vec{\lambda}_{r} \right|.cos\left( \theta\right);\left| \vec{\lambda}_{r_{b}} \right|=\left| \vec{\lambda}_{r} \right|.sin\left( \theta\right) & (2,3) \\ \mathrm{Rewriting}\left( 1 \right), & & \\ \vec{\lambda}_{r} & =\left| \vec{\lambda}_{r_{f}} \right|\hat{i}+\left| \vec{\lambda}_{r_{b}} \right|\hat{j} & (4) \\ \mathbf{Case 1}: & & \\ If, & \theta\in\left( 0,2\pi\right)-\left\{ 0,\frac{\pi}{2},\pi,\frac{3\pi}{2},2\pi\right\} & \\ then, & \left\{ \cos\left( \theta\right),sin\left( \theta\right) \right\}\in\left( -1,1 \right)\backslash\left\{ 0 \right\} & (5) \\ & & \\ Clearly, & \left| \vec{\lambda}_{r_{f}} \right|<\vec{\lambda}_{r};\left| \vec{\lambda}_{r_{b}} \right|<\vec{\lambda}_{r} & (6,7) \\ & & \\ Define, & & \\ \phi_{r} & =\left| \vec{\lambda}_{r_{f}} \right|\hat{i}+\left| \vec{\lambda}_{r_{b}} \right|\hat{j} & (8) \\ \left\| \phi_{r} \right\|_{2} & =\left| \vec{\lambda}_{r_{f}} \right|^{2}.\hat{i}^{2}+\left| \vec{\lambda}_{r_{b}} \right|^{2}.\hat{j}^{2}+2.\left| \vec{\lambda}_{r_{f}} \right|\hat{i}.\left| \vec{\lambda}_{r_{b}} \right|\hat{j} & (9) \\ & =\left( \left| \vec{\lambda}_{r_{f}} \right|+\left| \vec{\lambda}_{r_{b}} \right| \right)^{2} & (10) \\ & <\left( 2.\left| \vec{\lambda}_{r} \right| \right)^{2} & (11) \\ & <4.\left| \vec{\lambda}_{r} \right|^{2} & (11.1) \\ & <4.\left\| \vec{\lambda}_{r} \right\|_{2} & (11.2) \\ \left\| \vec{\lambda}_{r} \right\|_{2} & >\frac{1}{4}.\left\| \phi_{r} \right\|_{2} & (11.3) \\ \Rightarrow& \left\| \vec{\lambda}_{r} \right\|_{2}\neq\left\| \phi_{r} \right\|_{2} & (12) \\ & & \\ From (12) & We have a contradiction if we assume the rate constant for a reaction & \\ & is a vector rather a scalar quantity & \\ \mathbf{Case 2}: & & \\ If, & \theta\in\left\{ 0,\frac{\pi}{2},\pi,\frac{3\pi}{2},2\pi\right\} & \\ Then, & & \\ & \left\{ \cos\left( \theta\right),sin\left( \theta\right) \right\}\in\left\{ -1,1 \right\}\backslash\left\{ 0 \right\} & (13) \\ The p2-norm is, & & \\ \left\| \vec{\lambda}_{r} \right\|_{2} & =\left\{ \left\| \vec{\lambda}_{r_{f}} \right\|_{2},\left\| \vec{\lambda}_{r_{b}} \right\|_{2} \right\} & (14) \\ \vec{\lambda}_{r} & =\left\{ \sqrt{\left\| \vec{\lambda}_{r_{f}} \right\|_{2}},\sqrt{\left\| \vec{\lambda}_{r_{b}} \right\|_{2}} \right\} & (15) \\ & =\left\{ \left\{ 1,-1 \right\},\left\{ 1,-1 \right\} \right\} & (15.1) \\ Clearly, & & \\ \left| \vec{\lambda}_{r_{f}} \right| & =\left\{ 1,-1 \right\} & (16) \\ & =\left| \vec{\lambda}_{r_{b}} \right| & (16.1) \\ \Rightarrow& \left| \vec{\lambda}_{r_{f}} \right|.\left| \vec{\lambda}_{r_{b}} \right|\neq0 & (17) \\ Define, & & \\ & \beta\in\left\{ 1,-1 \right\}\backslash\left\{ 0 \right\} & \\ Then, & & \\ & \left| \vec{\lambda}_{r_{f}} \right|.\left| \vec{\lambda}_{r_{b}} \right|=\beta& (18) \\ & \left| \vec{\lambda}_{r_{f}} \right|=\beta.\left| \vec{\lambda}_{r_{b}} \right|^{-1} & (19) \\ From (12),(19) & The rate constant for a given reaction is a scalar rather a vector & \\ & \vec{\lambda}_{r}\equiv\lambda_{r} & \\ & ∎ & \end{matrix}$$

**Proof (T1)**:

$$\begin{matrix} \mathrm{For}\left\{ \mathbf{a},\mathbf{b} \right\}\in\mathcal{H} where \#\mathcal{H}=2 and \mathbf{a},\boldsymbol{b\in}\mathbb{R}^{\mathbf{I}} & \\ we have the partitions: & \\ \left\{ \mathbf{a,b} \right\}≝A_{2a} & (20) \\ \left\{ \begin{aligned} \left\{ . \right\},a\vee b \end{aligned} \right\}≝A_{2b} & (21) \\ & \\ \mathrm{For}\left\{ \mathbf{a,b,c} \right\}\in\mathcal{H} where \#\mathcal{H}=3 and \mathbf{a},\boldsymbol{b,c\in}\mathbb{R}^{\mathbf{I}} & \\ we have the partitions: & \\ \left\{ \mathbf{a,b,c} \right\}≝A_{3a} & (22) \\ \left\{ \begin{aligned} \left\{ .,. \right\},a\vee b\vee c \end{aligned} \right\}≝A_{3b} & (23) \\ & \\ \mathrm{For}\left\{ \mathbf{a,b,c,d} \right\}\in\mathcal{H} where \#\mathcal{H}=4 and \mathbf{a},\boldsymbol{b,c,d\in}\mathbb{R}^{\mathbf{I}} & \\ we have the partitions: & \\ \left\{ \mathbf{a,b,c,d} \right\}≝A_{4a} & (24) \\ \left\{ \left\{ \begin{aligned} \left\{ .,. \right\},\left\{ .,. \right\} \end{aligned} \right\} \right\}≝A_{4b} & (25) \\ \left\{ \begin{aligned} \left\{ .,.,. \right\},a\vee b\vee c\vee d \end{aligned} \right\}≝A_{4c} & (26) \\ & \\ \mathrm{For}\left\{ \mathbf{a,b,c,d,e} \right\}\in\mathcal{H} where \#\mathcal{H}=5 and \mathbf{a},\boldsymbol{b,c,d,e\in}\mathbb{R}^{\mathbf{I}} & \\ we have the partitions: & \\ \left\{ \mathbf{a,b,c,d,e} \right\}≝A_{5a} & (27) \\ \left\{ \begin{aligned} \left\{ \left\{ .,. \right\},\left\{ .,. \right\} \right\},a\vee b\vee c\vee d\vee e \end{aligned} \right\}≝A_{5b} & (28) \\ \left\{ \begin{aligned} \left\{ \left\{ .,. \right\},\left\{ .,.,. \right\} \right\} \end{aligned} \right\}≝A_{5c} & (29) \\ & \\ We can exclude A_{2b},A_{3b},A_{4c},A_{5b} & \\ We will retain A_{2a},A_{3a},{A_{4a},A}_{4b},A_{5a},A_{5c} & \\ & \\ \Theta≝\left\{ \begin{aligned} 1 iff \#\mathcal{H}=\left\{ 2,3 \right\} \\ \geq2 iff \#\mathcal{H}\geq4 \end{aligned} \right. & (30) \\ =1+\sum_{t=2}^{t=\#\mathcal{H}_{u}-2} \binom{\#\mathcal{H}_{u}}{t} & (31) \\ ∎ & \end{matrix}$$

**Proof (C1):**

$$\begin{matrix} \begin{matrix} \mathcal{V}_{u} & =N\left( \mathcal{S}_{\mathcal{p}_{z}} \right)\cup\mathcal{G}_{u}\cup\mathcal{H}_{u}\cup{\bar{\mathcal{H}}}_{u}\cup\mathcal{L}_{u} & (32) \\ & ≝\left( N\left( \mathcal{S}_{\mathcal{p}_{z}} \right)\cup\mathcal{G}_{u}\cup\mathcal{H}_{u} \right)\cup{\bar{\mathcal{H}}}_{u}\cup\mathcal{L}_{u} & (33) \\ & =\mathcal{A}_{u}\cup{\bar{\mathcal{H}}}_{u}\cup\mathcal{L}_{u} & (34) \\ & & \\ \#\mathcal{V}_{u} & =\#\mathcal{A}_{u}+\#{\bar{\mathcal{H}}}_{u}+\#\mathcal{L}_{u} & (35) \\ & >\#\mathcal{A}_{u} & (36) \\ & ∎ & \end{matrix} \end{matrix}$$

**Proof (C4)**:

$$\begin{matrix} \mathrm{From}\mathbf{(C1-C3)}, & & \\ & T_{u}\mathcal{<O}\left( \left. 2^{\#\mathcal{V}_{u}} \right..\#\mathcal{V}_{u} \right) & (37) \\ & ∎ & \end{matrix}$$

**Proof (T2):**

$$\begin{matrix} & For the reaction-specific sequence from a row, & \\ & \left( a_{u_{i_{k}}} \right)_{k=1,2\ldots K}|a_{u_{i_{k}}}\mathbb{\in R\cap}\left( -\infty,\infty\right) \forall k & (38) \\ & where K\mathbf{=}\mathrm{cols}\left( \mathcal{A}_{u} \right) & \\ & & \\ & We redefine and rewrite each term as, & \\ & a_{u_{i_{k}}}≝\left( -1 \right)^{2k}.a_{u_{i_{2k}}}\mathrm{iff}a_{u_{i_{k}}}>0 & (39) \\ & a_{u_{i_{k}}}≝\left( -1 \right)^{2k-1}.a_{u_{i_{2k-1}}}\mathrm{iff}a_{u_{i_{k}}}<0 & (40) \\ & & \\ & Let P be the number of positive terms & \\ & Let L be the number of negative terms & \\ & & \\ \phi_{u_{i}} & \mathbf{=}\sum_{k=1}^{k=K} a_{u_{i_{k}}} & (41) \\ & \mathbf{=}\sum_{k=1}^{k=K} \left( \left( -1 \right)^{2k}.a_{u_{i_{2k}}}\mathbf{+}\left( -1 \right)^{2k-1}.a_{u_{i_{2k-1}}} \right) & (41.1) \\ & \mathbf{=}\sum_{k=1}^{k=P+L} \left( \left( -1 \right)^{2k}.a_{u_{i_{2k}}}\mathbf{+}\left( -1 \right)^{2k-1}.a_{u_{i_{2k-1}}} \right) & (41.2) \\ & \mathbf{=}\sum_{k=1}^{k=P} a_{u_{i_{k}}}\mathbf{+}\sum_{k=1}^{k=L} \left( -1 \right).a_{u_{i_{k}}} & (41.3) \\ & \mathbf{=}\sum_{k=1}^{k=P} a_{u_{i_{k}}}\mathbf{+}\left( -1 \right).\sum_{k=1}^{k=L} a_{u_{i_{k}}} & (41.4) \\ & \mathbf{≝}\phi_{u_{i_{k}}}+{\left( -1 \right).\phi}_{u_{i_{k}}} & (42) \\ & ∎ & \end{matrix}$$

**Proof (T3):**

$$\begin{matrix} If K=\#cols\left( \mathcal{A}_{u} \right)\mathrm{and} & \\ P≝Number of positive terms & (43) \\ L≝Number of negative terms & (44) \\ s.t. P+L=K & (45) \end{matrix}$$

$$\begin{matrix} & if, a_{u_{i_{k}}}>0 \forall k & (46) \\ & then after u=U>M iterations and from \left( \mathbf{T2} \right) & \\ & P=K and L=0 & (47,48) \\ & & \\ & and, & \\ & \phi_{u_{i_{k}}}>1 & (49) \\ & \mathrm{and}{\left( -1 \right).\phi}_{u_{i_{k}}}=0 & (50) \\ & & \\ & \Rightarrow\phi_{u_{i}}>1 & (51) \\ & \exists g:\phi_{u_{i}}\mapsto y=\phi_{u_{i}}\mathbb{\in R\cap}\left( 1,\infty\right) & (52) \\ & & \\ & if, a_{u_{i_{k}}}<0 \forall k & (53) \\ & then after u=U>M iterations and from \left( \mathbf{T2} \right) & \\ & P=0 and L=K & (54,55) \\ & & \\ & and, & \\ & {\left( -1 \right).\phi}_{u_{i_{2k-1}}}<-1 & (56) \\ & \mathrm{and}\phi_{u_{i_{2k}}}=0 & (57) \\ & & \\ & \Rightarrow\phi_{u_{i}}<-1 & (58) \\ & \exists g:\phi_{u_{i}}\mapsto y=e^{\phi_{u_{i}}}\mathbb{\in R\cap}\left( 0,1 \right) & (59) \\ & & \\ & if,\left\{ \left( a_{u_{i_{k}}}>0 \right) \vee\left( a_{u_{i_{k}}}<0 \right) \right\} \forall k & (60) \\ & then after u=U>M iterations & \\ & P\neq0 and L\neq0 & (61,62) \\ & and, & \\ & & \\ a) & \left| \phi_{u_{i}} \right|\gg0 & (63) \\ & \Rightarrow\left( \phi_{u_{i}}\gg0 \right)\mathrm{or}\left( 0\gg\left( -1 \right).\phi_{u_{i}} \right) & (64) \\ & & \\ & Choose P=1 (number of of positive terms) & (65) \\ & and L=K-1 & (66) \\ & & \\ & Clearly, & \\ & \left( \phi_{u_{i_{k}}}>1 \right)\wedge\left( {\left( -1 \right).\phi}_{u_{i_{k}}}<-1 \right) & (67) \\ & \mathrm{and} & \\ & g\left( \phi_{u_{i_{2k}}} \right)+g\left( \left( -1 \right).\phi_{u_{i_{2k-1}}} \right) (From \mathbf{T2}) & (68) \\ & =y_{1}+y_{2} & (68.1) \\ & >y_{1} (Since y_{1}\mathbb{\in R\cap}\left( 1,\infty\right)\mathrm{and}y_{2}\mathbb{\in R\cap}\left( 0,1 \right)) & (68.2) \\ & \mathbb{\in R\cap}\left( 1,\infty\right) & (68.3) \\ & & \\ b) & \phi_{u_{i}}\approx0 (From \mathbf{T2}) & (69) \\ & \Rightarrow\phi_{u_{i_{2k}}}=\phi_{u_{i_{2k-1}}} & (70) \\ & \Rightarrow P\approx L (For a sufficiently large u=U>m) & (71) \\ & \exists g:\phi_{u_{i}}\mapsto y=e^{\phi_{u_{i}}}\mathbb{\in R\cap}\left\{ 1 \right\} & (72) \\ & ∎ & \end{matrix}$$

**Proof (C5):**

$$\begin{matrix} From Defs.\left( 22 \right), & \mathrm{if}a_{i_{f}}\in\mathcal{F}_{i} for u=U>M & \\ then, & a_{i_{k}}\in\mathcal{F}_{i} & (73) \\ \Rightarrow& a_{i_{k}}\notin\mathcal{B}_{i} & (74) \\ From Def.\left( 23 \right), & \mathrm{if}a_{i_{b}}\in\mathcal{B}_{i} for u=U>M & \\ then, & a_{i_{k}}\in\mathcal{B}_{i} & (75) \\ \Rightarrow& a_{i_{k}}\notin\mathcal{F}_{i} & (76) \\ From (73-76), & & \\ it follows, & & \\ & \mathrm{if} \left\{ \mathcal{F}_{i},\mathcal{B}_{i} \right\}\neq\emptyset& \\ & \mathrm{then}\mathcal{F}_{i}\cap\mathcal{B}_{i}=\emptyset& (77) \\ & & \\ Since, & \mathcal{E}_{i}≝\mathcal{F}_{i}\cup\mathcal{B}_{i} & (78) \\ it follows, & & \\ & a_{i_{e}}\in\left( \mathcal{F}_{i}\cup\mathcal{B}_{i} \right) & (79) \\ & =\left( a_{i_{e}}=a_{i_{f}}\in\mathcal{F}_{i} \right)\cup\left( a_{i_{e}}=a_{i_{b}}\in\mathcal{B}_{i} \right) & (79.1) \\ & =\left( a_{i_{e}} \right)_{e=1,2\ldots\#\mathcal{F}_{i}+\#\mathcal{B}_{i}} & (79.2) \\ & =\left( \left( -1 \right)^{2e}.a_{2e}+\left( -1 \right)^{2e-1}.a_{2e-1} \right)_{e=1,2\ldots\#\mathcal{F}_{i}+\#\mathcal{B}_{i}} & (79.3) \\ with the sum, & & \\ & \sum_{e=1}^{e=\#\mathcal{F}_{i}+\#\mathcal{B}_{i}} \left( \left( -1 \right)^{2e}.a_{2e}+\left( -1 \right)^{2e-1}.a_{2e-1} \right) & (80) \\ & =\sum_{e=1}^{e=\#\mathcal{F}_{i}} a_{i_{f}}+\left( -1 \right).\sum_{e=1}^{e=\#\mathcal{B}_{i}} a_{i_{b}} & (80.1) \\ & =\phi_{i_{\mathcal{F}}}+\left( -1 \right).\phi_{i_{\mathcal{B}}} \left( From T2,T3 \right) & (80.2) \\ & ≝\phi_{i_{\mathcal{E}}} & (81) \\ & =\sum_{e=1}^{e=\#\mathcal{E}_{i}} a_{i_{e}}{for the sequence \left( a_{i_{e}} \right)}_{e=1,2\ldots\#\mathcal{E}_{i}} & (81.1) \\ & ∎ & \end{matrix}$$
